# Contextual influences on mental health in pharmacy education: a comparative study across higher- and lower-middle-income settings

**DOI:** 10.1080/10872981.2026.2756743

**Published:** 2026-11-05

**Authors:** Hamza Saeed, Wendy Thompson, Katherine M. White, Judith Singleton

**Affiliations:** a School of Clinical Sciences, Faculty of Health, Queensland University of Technology, Brisbane, Australia; b School of Psychology and Counselling, Faculty of Health, Queensland University of Technology, Brisbane, Australia

**Keywords:** Student mental health, pharmacy students, mental health challenges, help-seeking behaviour, academic stress

## Abstract

**Introduction:**

Student mental health has emerged as a major challenge for health professional training worldwide, particularly among students enrolled in often demanding professional programmes such as pharmacy. Although universities increasingly recognise the importance of student wellbeing, less is known about how pharmacy students’ mental health experiences are shaped by differing institutional and socio-cultural contexts. This study examines the contextual influences shaping pharmacy students’ mental health and help-seeking behaviours across two contrasting pharmacy education contexts: Australia, a high-income country, and Pakistan, a lower-middle-income country.

**Methods:**

A qualitative design was employed using semi-structured interviews with 18 pharmacy students from universities in both countries. An inductive thematic approach was adopted to identify broader thematic patterns across two different cultural and educational contexts.

**Results:**

The analysis revealed several interconnected themes influencing students’ mental health including academic stressors; personal experiences and well-being; family, peer and social expectations; culture, stigma and attitudes to mental health; awareness and knowledge; coping strategies and support mechanisms; help-seeking behaviour and barriers; and institutional and system-level support. While students across both contexts reported significant academic pressure and emotional strain, notable differences emerged in the availability, accessibility, and perceived responsiveness of institutional mental health support, as well as in cultural attitudes towards help-seeking.

**Conclusion:**

By examining these experiences across contrasting pharmacy education contexts, this study provides cross-national insights into how institutional environments and socio-cultural factors shape pharmacy students’ mental health experiences. The findings highlight importance of developing accessible and contextually responsive strategies to support student wellbeing within pharmacy education.

## Introduction

Poor mental health among students undertaking health professional training is increasingly recognised as a significant global challenge [1]. University students face a range of mental health challenges, including depression and anxiety [2], which may be associated with academic, financial, and social pressures [3,4]. A study involving first-year university students across eight countries found that 31.4% screened positive for at least one mental disorder in the preceding year [5]. The COVID-19 pandemic further affected students’ mental health, introducing additional challenges such as loneliness, remote learning, and uncertainty [6,7]. Although these challenges impact all university students, those enrolled in pharmacy programmes face distinctive academic and professional pressures warranting focused consideration.

Stress, anxiety, and depression are common mental health concerns among pharmacy students, particularly female students [8,9]. A study comparing pharmacy and medical students reported higher rates of clinically significant anxiety among pharmacy students (21% versus 11%), who were also less likely to seek help from university counselling services [8]. Another study found that more than 25% of pharmacy students scored in the high-severity range for depression, generalised anxiety, academic distress, and eating concerns [9]. The pharmacy curriculum presents substantial academic challenges, including laboratory tasks, practical training, and demanding assessments, which may contribute to stress and difficulties with time management [9,10]. A study involving 304 pharmacy students revealed that 30% of participants self-identified as experiencing clinically significant anxiety symptoms, while 22% reported clinically significant depressive symptoms [11]. These factors along with stigma surrounding mental health, can create significant barriers for pharmacy students accessing help and support [12]. The mental health of pharmacy students is crucial as the responsibilities of pharmacists have evolved significantly in recent years [13]. The role of the pharmacist has transformed from a traditional focus on dispensing medicines to a more patient-centred and collaborative model of care [14]. Pharmacists now actively contribute to medication management, chronic disease prevention, immunisation, and health promotion within multidisciplinary teams [15]. It is important that pharmacy students are well supported to manage the stressors impacting their mental health as they will graduate into a workforce required to handle the growing complexities of these expanded roles.

Mental health conditions frequently remain unrecognised or unaddressed because university students often hesitate to seek help from mental health providers [16]. Instead, many students rely upon informal coping strategies such as peers [17]. This strategy may neglect underlying conditions, thereby increasing the risk of academic failure, social withdrawal, or chronic mental health issues in the future [18]. Numerous institutional and systemic barriers including stigma, inconsistent policies, and suboptimal service quality, hinder access to mental health support [5,19]. These barriers appear to be more pronounced in low and middle income countries (LMICs), where institutional mental health support within universities is often insufficient or absent. Research conducted in high income countries (HICs) provided a foundational understanding of the distinct mental health challenges encountered by students and played a crucial role in the establishment of university counselling services as the main support system [20]. Addressing the mental health requirements of students in healthcare professions necessitates a comprehensive strategy that encompasses support from peers, institutional support, and personal coping mechanisms [21–23]. These challenges highlight the importance of considering how differences in educational environments and available support may shape pharmacy students’ mental health experiences and help-seeking behaviours.

Pharmacy education in Australia and Pakistan aims to prepare students for professional pharmacy practice; however, the educational systems and training pathways differ. In Australia, pharmacy programmes are accredited by the Australian Pharmacy Council (APC), and registration requires completion of an APC-accredited degree, usually a four-year Bachelor of Pharmacy (Honours), followed by a supervised internship of 1,575 hours and successful completion of registration examinations [24,25]. In Australian pharmacy education, student wellbeing is supported both through accreditation expectations and through curriculum content that explicitly addresses mental health and resilience [24]. Australian pharmacy programmes place a strong emphasis on practical skill development through placements and experiential learning, including activities such as vaccination training and medication management [26]. Students are also encouraged to seek employment in community pharmacies during their studies, often from their second year, enabling them to gain practical experience alongside their academic training.

In Pakistan, pharmacy education is delivered through a five-year Doctor of Pharmacy (PharmD) programme accredited by the Pharmacy Council of Pakistan. Graduates are eligible for registration upon completion of the degree and registration with the Council [27]. Student wellbeing and mental health support receive limited explicit attention within descriptions of pharmacy education and educational practice in Pakistan. Although practical training forms part of the curriculum, pharmacy-related employment during study is less common and is not routinely integrated into the educational pathway [28]. Pharmacy students in Pakistan tend to focus primarily on academic coursework and university-based training [28]. While pharmacy education in both countries is academically demanding, differences in programme structure, opportunities for workplace exposure, and access to student support services may influence students' educational experiences and how they respond to academic and personal challenges.

Although pharmacy education in Australia and Pakistan shares the common goal of preparing students for professional practice, students undertake their training within different educational and sociocultural contexts that may influence perceptions of mental health, stigma, emotional disclosure, family expectations, and help-seeking. Understanding these contextual influences is important because mental health experiences cannot be assumed to have equivalent meanings across different cultural settings.

Research from HICs, including Australia, has increasingly examined student well-being and mental health support in university environments. In contrast, evidence from LMICs such as Pakistan, is limited and inconsistent with little research exploring mental health challenges in pharmacy students specifically. To address this research gap, this study aimed to explore the experiences and perceptions of Australian and Pakistani undergraduate pharmacy students regarding mental health and help-seeking behaviours. Comparing Australian and Pakistani pharmacy students’ experiences and perceptions is useful due to the significant differences in cultural attitudes, health infrastructure, and the availability of mental health resources. This comparison offers insights into the influence of socioeconomic and cultural contexts on mental health perceptions and help-seeking behaviours in pharmacy students.

## Methods

### Study design

This qualitative study used semi-structured interviews which allowed pharmacy students to talk about their experiences and perceptions in their own words, enabling a deeper understanding of the academic, cultural, social, and institutional factors that shape their wellbeing, and their attitudes towards seeking help. Ethical approval was obtained from the Queensland University of Technology Human Research Ethics Committee (Approval number: 8885). The study was conducted in accordance with the ethical principles outlined in the Declaration of Helsinki.

### Study setting

Participants were undergraduate pharmacy students enrolled in Bachelor programs at universities in Australia (*n* = 8) and Pakistan (*n* = 10). Convenience sampling was used to recruit students, and no participant attrition was reported. Recruitment was undertaken via university portals/networks, social media, and student associations, with assistance from academic leads and course coordinators/program lead.

### Data collection

A topic guide was developed, informed by the existing literature and refined through input from the research team [29]. After initial piloting with volunteer students, the topic guide was refined for clarity through minor revisions to question wording and the addition of prompts. Interview questions comprised initial demographic information questions followed by open-ended questions and follow-up prompts exploring participants’ experiences of mental health challenges and help-seeking behaviours (Supplementary File S1). Interviews were conducted independently in a private online setting via Zoom lasting between 30 to 45 minutes. Recruitment continued until thematic saturation was reached [30] and no new insights emerged. At the beginning of each session, the first author introduced himself and explained the study. Verbal consent was obtained and each participant was assigned a unique ID code (*P* for Pakistan and *A* for Australia and interview number). Interviews were both audio and video recorded (to allow observation of non-verbal cues in real time), with only the audio recordings retained for transcription and analysis. As a small token of appreciation, Australian students received a AUD$20 voucher while Pakistani students received a PKR2000 voucher. Interviews were conducted between April and September 2025 in Australia and between April and July 2025 in Pakistan.

### Data analysis

The purpose of this study was to identify broader thematic patterns across two different cultural and educational contexts, so an inductive thematic approach [31] was undertaken for data analysis. This approach facilitated the generation of themes directly from participants’ narratives rather than applying pre-determined theoretical categories. To ensure transparency and rigour in reporting, the study adhered to the consolidated criteria for reporting qualitative research (COREQ) checklist [32] (Supplementary File S2). For Pakistani participants, interviews conducted in Urdu were first transcribed verbatim, and then translated into English. All translations into English were independently reviewed by a second researcher to ensure accuracy. For Australian participants, Zoom’s automated transcription function was used as an aid, with all transcripts manually reviewed and corrected against the audio recordings by a second reviewer to ensure accuracy.

Thematic analysis of the data was undertaken [31]. The first author undertook multiple readings of the transcripts to achieve familiarity with the data before manual coding in NVivo (Version 14) [33] commenced. The first step utilised an open coding approach, as described by Saldaña [34], in which initial codes were generated to capture key concepts reflected in participants’ transcripts. The second step involved axial coding [34], where linkages and the relationships between the open codes were studied in greater detail. Next, the first author explored how codes related to one another and to the broader phenomena under study. Related codes were merged into sub-themes, which were then synthesised into overarching main themes that showed the main patterns in the data. A subset of transcripts was separately reviewed and coded by a second author to validate and provide peer feedback on the codes. These themes were reviewed against the coded data and the dataset as a whole before being refined, named, and clearly defined. The final analysis was then reviewed by the fourth author and any differences in opinion were resolved through discussion.

In addition to following Braun and Clarke's approach [31] to thematic analysis, the comparative analysis was informed by published guidance for conducting cross-cultural qualitative research, which emphasises preserving linguistic, educational, and sociocultural context while identifying both shared and context-specific experiences [35]. Australia and Pakistan were not treated as directly equivalent settings; rather, analysis recognised that participants' accounts were shaped by broader social, cultural, familial, institutional, and educational influences. Interview transcripts from each country were initially analysed independently, with coding and subtheme development undertaken separately to preserve the contextual meaning of participants' accounts. Country-specific subthemes were then compared iteratively through discussion within the research team to identify conceptually similar experiences across both datasets. Subthemes reflecting the same underlying phenomenon were synthesised into shared overarching themes, while differences in how these experiences were understood and described informed interpretation. Particular attention was given to factors such as family expectations, stigma, help-seeking norms, support systems, and educational environments to ensure that common themes reflected shared experiences without assuming they carried the same meaning across both countries. This approach enabled the identification of shared overarching themes while preserving the contextual meaning of country-specific experiences during interpretation. The overall analytical process is illustrated in Figure 1.

**Figure 1.**
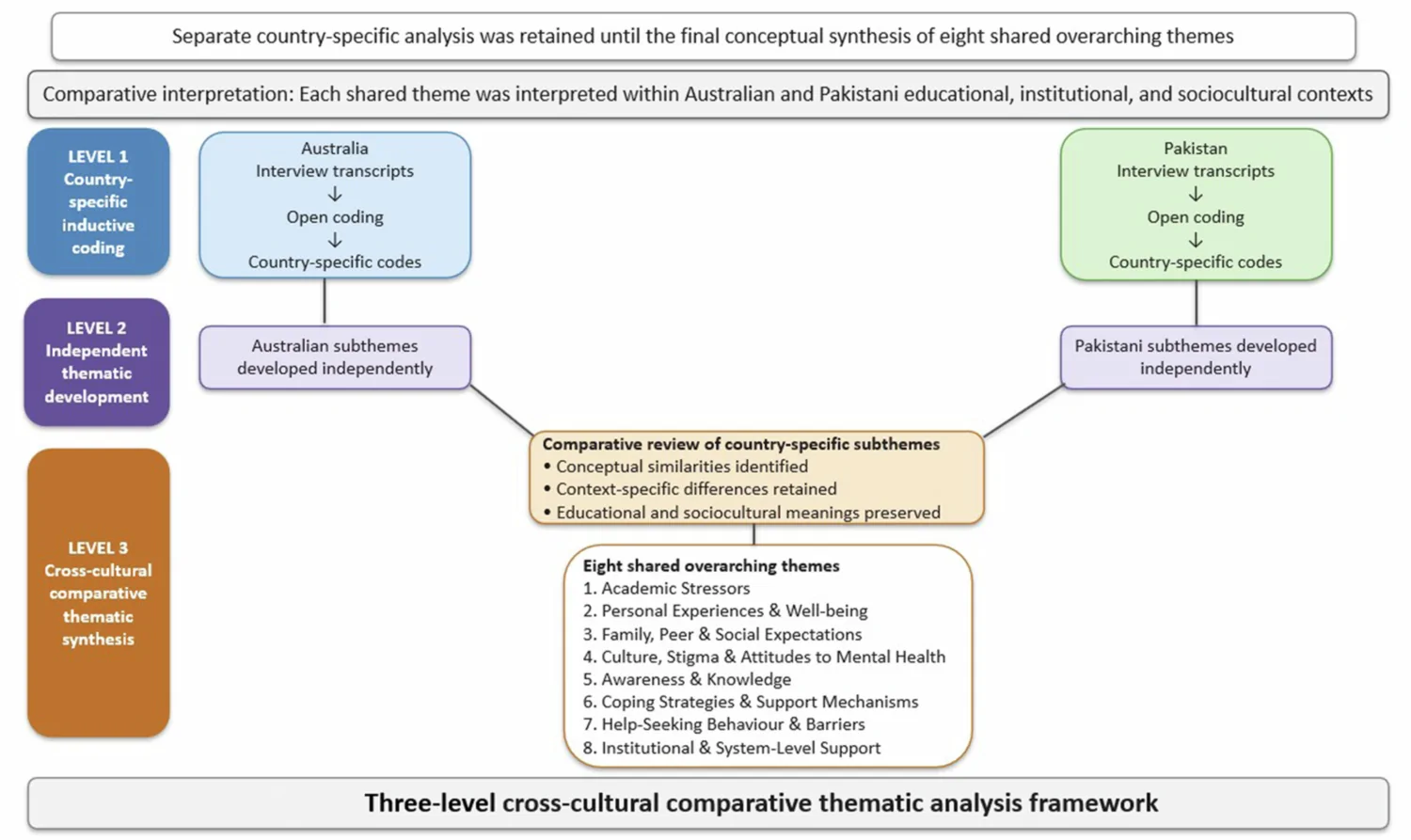
Three-level cross-cultural comparative thematic analysis framework.

### Bias

To minimise selection bias, participants were recruited through multiple channels and from different universities within each country. Numerous steps were taken to ensure the trustworthiness of the research process. The analytical approach was established *a priori* and agreed upon by the research team. Weekly meetings were held to discuss coding decisions, challenges, and to resolve any differences in analysis by consensus. An audit trail was maintained to document coding changes, analytical decisions, and reflections throughout the study. The COREQ checklist guided the reporting process [32]. In line with the criteria proposed by Chiovitti and Piran, rigour was assessed in terms of credibility, auditability, and fittingness [36]. To enhance credibility, the analysis adhered closely to the data through repeated readings, inductive coding, and the use of participants’ own words (in vivo codes) to preserve meaning. Review of a subset of transcripts by the third author further strengthened credibility by providing validation of the coding process. Auditability was ensured by providing extensive documentation of participant recruitment, coding and thematic process, enabling another researcher to follow the steps and reach comparable conclusions. Fittingness was addressed by situating the findings within the wider literature and by clearly outlining the context, participant characteristics, and scope of the study. Reflexivity was maintained throughout the study. The interviewer had no prior relationship with the participants before recruitment. No repeat interviews were conducted, and no field notes were taken during or after the interviews. Interview transcripts were not returned to participants for review or correction, and participants were not involved in reviewing or validating the final themes and findings. The first author drew on his previous experience as a male pharmacist and pharmacy lecturer in Pakistan, which facilitated rapport and communication with Pakistani participants through a shared language and familiarity with the educational and cultural context. His current role as a doctoral researcher and sessional academic in Australia, together with ongoing discussions with his supervisors, who are registered Australian pharmacists, also provided an understanding of the Australian pharmacy education environment. Throughout the study, the research team reflected on how these experiences could shape data collection and interpretation. During analysis, discussions focused on ensuring that participants' accounts were interpreted within the context in which they were shared, rather than through assumptions based on the researchers’ own experiences. This approach supported the preservation of country-specific meanings before conceptually similar subthemes were brought together into shared overarching themes.

## Results

In Australia, all 18 institutions identified as offering undergraduate pharmacy programmes were approached for participant recruitment, resulting in eight participants from five institutions. In Pakistan, 16 institutions across multiple provinces were approached, resulting in ten participants from seven institutions. Overall, 18 undergraduate pharmacy students participated in the study (Table 1).

**Table 1.** Summary of participant characteristics.

Characteristic	Australia (*n* = 8)	Pakistan (*n* = 10)
**Age (years)**		
Mean ± SD	20.50 ± 1.77	22.50 ± 2.12
**Year of study, *n* (%)**		
First year	3 (37.5%)	1 (10.0%)
Second year	1 (12.5%)	2 (20.0%)
Third year	2 (25.0%)	1 (10.0%)
Fourth year	2 (25.0%)	1 (10.0%)
Fifth year	N/A	5 (50.0%)
**Employment status, *n* (%)**		
Part-time	6 (75.0%)	2 (20.0%)
Casual work	2 (25.0%)	1 (10.0%)
Unemployed	0 (0.0%)	7 (70.0%)
**Living arrangements, *n* (%)**		
With parents	5 (62.5%)	0 (0.0%)
With extended family	0 (0.0%)	7 (70.0%)
With partner	1 (12.5%)	0 (0.0%)
Shared house	2 (25.0%)	0 (0.0%)
Private accommodation	0 (0.0%)	3 (30.0%)

Note: Percentages use the total number of participants within each country. Categories reproduce those recorded in the original participant data.

Abbreviation: SD, sample standard deviation.

The cross-cultural comparative analytical process resulted in eight shared overarching themes derived from independently developed country-specific subthemes (Figure 1). The analysis focused on identifying codes in participants’ interviews regarding the challenges that influence their mental health and help-seeking behaviours. From the coding process, key sub-themes and themes emerged from the data. Tables 2 and 3 show sub-themes and key themes, together with representative quotations from Australian and Pakistani participants respectively. The manual coding trees for the Australian and Pakistani participants are presented in Supplementary Files S3 and S4 respectively.

**Table 2.** Representative quotations, sub-themes and key themes of Australian participants.

Representative Quotations	Sub-themes	Themes
“Workload has been intense from the very start. First year, there was a statistics unit with a final assignment, that was daunting—straight out of high school to that. Another time…………. I had an exam on Wednesday, an assignment due Friday, I was working the weekend, and then an exam on Monday” (A5). “OSCEs time is the most stressful time” (A7).	Workload, Exams and Assessment Pressure	Academic Stressors
“I expected university to be a bit easy, but I was not prepared for the overwhelming amount of content…………. The material is not necessarily difficult—it is the quantity. Also, multiple 3-hour practicals for each course take a lot of time, and then there is more work at home. All this workload is draining” (A6).	Time Pressure, Clashing Demands, and Work–Study Balance	
“I also started university in a different city and moved to make it easier. Moving away from home was hard to adjust to, but I am doing better now” (A7).	Transition and Academic Adjustment	
“There has been a significant increase in stress and anxiety since I began doing pharmacy. I had a history of anxiety that had settled, but it has flared up and now affects both my studies and personal life” (A6).	Social Life, Self-Care and Relationships	Personal Experiences and Well-being
“The main challenge is financial. I come from a low- to medium-income household, and my parents cannot help with study fees, including mandatory placements” (A8).	Financial Stress, Work and Structural Constraints	
“There was a time in my studies when I felt conflicted with myself, also had prolonged sadness, confusion, loss of direction. I struggled to get up or do what I used to enjoy” (A8).	Emotional Burden and Psychological Impact	
“My mum is not strict, but she pushes for passing everything and good grades. I want to make my family proud, that adds pressure and contributes to stress and anxiety” (A6).	Family Expectations, Pressures and Ambitions	Family, Peer and Social Expectations
“I keep the house clean, take care of the dog, and make most dinners since I am usually home earlier than my parents. It influences timing…….” (A4).	Family Responsibilities and Household Roles	
“I also find myself comparing to friends in other degrees or the same degree” (A6).	Peer Relationships, Comparison and Identity	
“In Australian culture there is definitely a generational difference. Tell an 80-year-old you are seeing a therapist, and they might see it as shameful. To people our age, if you say you are on antidepressants, they might often say, ‘Me too’…………” (A5).	Cultural Attitudes, Stigma, and generational differences in attitudes	Culture, Stigma and Attitudes to Mental Health
“People say mental health is important, but it is not really treated the same as physical health. It is often more of a throwaway statement………... It feels a bit foreign, something different, not part of normal health conversations” (A3).	Openness and Comfort Discussing Mental Health	
“Awareness is growing. At hospital where I work, mental health is well supported. I can identify when classmates are struggling—for example, one isolates herself completely, studies excessively, and admitted she feels lonely and stressed” (A2).	Awareness of Peers’ Mental Health and Proactive Support	Awareness and Knowledge
“In high school we did Teen Mental Health First Aid—recognising signs/symptoms and what to do” (A7).	Training, Programs and Mental Health Literacy	
“Professional help—counsellors/psychologists—because they have tools and training” (A6). “Friends are a big support. I also think helplines like Lifeline are great—they are free, anonymous, and easy to access without having to commit to a formal appointment” (A3).	Professional and informal support	Coping Strategies and Support Mechanisms
“Both professional and informal, depending on the issue. Interpersonal, relationship, and study-related problems are usually addressed through professional support (including couples therapy). Familial issues are often handled within the family” (A8).	Self-Directed, Lifestyle and Task-Oriented Coping	
“I assess risk—if there is any possibility of harm to myself or others, or that intrusive thoughts could push unhealthy behaviour, I would reach out immediately. My therapist would be my first point of contact” (A4). “Last year I felt…… anxious about work and study. Recognising I was not feeling like my usual self, it pushed me to seek help. I went to a GP, did online counselling, and took an antidepressant for about a month” (A7).	Help-Seeking Threshold, Decision Rules, and Sources of Help	Help-Seeking Behaviour and Barriers
“I did seek help but received none, so now I keep things to myself” (A2).	Barriers and Negative Experiences of Help-Seeking	
“Good—short-wait counselling, a 24/7 line, and temporary adjustments through the mental health support group” (A1). “…... Some of my friends have complained about the long wait time for in-person sessions” (A4).	Views on University Support and Desired Improvements	Institutional and System-Level Support
“Pharmacy (course) is very hard, and the stress is not discussed enough. Many are overwhelmed and mental health is affected….” (A6).	Intensity of Pharmacy Course	

**Table 3.** Representative quotations, sub-themes and key themes of Pakistani participants.

Representative Quotations	Sub-themes	Themes
“Going to university 8 am to 4 pm, five days a week is the most difficult part for me…….” (P2).	Workload and Time Pressure	Academic Stressors
“In vivas you do not know what will be asked, external examiner is not much familiar with what core topics have been taught, so this uncertainty creates stress” (P5).	Exams and Assessments	
“Starting university in 2020 during COVID—everything was online. As a first-semester student, there was no proper guidance, lectures were hard to follow, and my GPA suffered” (P6).	External Disruptions and Personal Consequences	
“I do struggle with daily headaches. It happens almost every day due to long study hours, and it affects me a lot” (P1).	Health and Functioning	Personal Experiences and Well-being
“Due to emotional drain, I felt low and somewhat depressed. Then I worked hard, improved each term and recovered” (P6).	Self-Reflection and Emotional Well-being	
“No special responsibilities—family wants me to focus on studies” (P7).	Family Pressures and Responsibilities	Family, Peer and Social Expectations
“…. Seeing peers excel in community work, sports, research, made me fear “Will I graduate and still be unknown here?” The anxiety of that troubles me” (P9).	Peer Pressure and Competition	
“Academically I managed, but personal financial crises created imbalance. I sometimes felt anxious and isolated. Over time I learned to control my anxiety” (P9).	Financial and Structural Barriers	
“Mental health is largely stigmatised/taboo, people prioritise physical health and may make fun if you raise mental-health concerns” (P8).	Stigma and Judgement	Culture, Stigma and Attitudes to Mental Health
“I am not comfortable. In our society, people judge you if you share personal struggles, especially about mental health. So, I prefer not to discuss it” (P1).	Comfort Levels	
“Mixed. Some treat it as a medical issue that needs management; others view it primarily through a religious lens and don’t accept it as a health condition” (P4).	Barriers and Variation in Attitudes	
“There is awareness, but it is uneven. People know terms like depression or anxiety yet understanding of what they really mean and how to respond is limited” (P3).	Awareness Gaps	Awareness and Knowledge
“Universities in Pakistan need much more awareness work—students face anxiety/depression but rarely talk about it” (P8).	Role of Education	
“I think I am aware. One pharmacy friend has panic attacks, especially around exams, and attends counselling. Early on people misread it as lack of religiosity; later they understood it is medical issue” (P4).	Personal Awareness and Reflection	
“First, I would discuss with family, then friends. They are closest and understand me…….” (P7).	Family Support Systems	Coping Strategies and Support Mechanisms
“Trusted close friends. Some teachers are approachable on a personal level, but there is no formal university support system that I am aware of” (P5).	Peer and Social Support	
“I had some mental health problems before, for which I did avail professional psychiatrist service………” (P10).	Professional Help	
“Faith-based coping, reciting Quran calms my anxiety and helps me through tough times” (P6).	Self and Creative Coping	
“Fear of family/friends finding out and attaching a label; social stigma. Finances can also be a barrier” (P5).	Financial and structured barrier	Help-Seeking Behaviour and Barriers
“Stigma—fear that society will know I am taking therapy” (P3).	Stigma and Judgement	
“Currently, zero (mental health support available). At orientation we were told a psychologist would be appointed; it has not happened yet………” (P10).	Systemic Barriers	Institutional and System-Level Support
“Not institutionally—no formal mental-health service. A couple of teachers are open to discussion informally” (P5).	University Environment	
“.…. Greater visibility and supportive services could make a real difference” (P8).	Counselling Infrastructure	
“Reduce course load/credit burden—the content and credits are heavy and drive stress. Exam formats and presentations vary by teacher. A broader structural support would help, but curriculum and exam control sits with departments” (P4).	Curriculum and Assessment Design	

### Theme 1: academic stressors

Academic stressors were among the most reported challenges faced by pharmacy students across both countries. Workload and time pressures were frequently described as major contributors to stress. For example, one participant noted, *“Heavy syllabus, so many subjects to cover in a short time, and draining lab work causes difficulty in managing time”* (P8). Participants also expressed concerns about external disruptions, such as the COVID-19 pandemic, which further contributed to their academic stress. Pakistani participants also reported feeling stressed due to the unclear examination process (Table 3) and Australian participants reported stress related to Objective Structured Clinical Examinations (OSCEs) which assess clinical competencies through a series of structured, simulated clinical stations involving role-play scenarios, with students’ performance evaluated by an examiner (Table 2).

### Theme 2: personal experiences and well-being

Personal experiences and well-being emerged as a salient theme across interviews with pharmacy students in both Australia and Pakistan. Participants described how psychological and physical impacts, as well as relationships and social life, shaped their lived experience and overall well-being. One participant stated, *“Last year I felt alone and overwhelmed—anxious about work, study load, and assignments which affected my personal life negatively”* (A7). Physical ailments also affected their overall well-being, further highlighting the interconnected nature of mental and physical health. Many participants prioritised academic commitments over their social life. Participants from Australia commonly reported these experiences (Table 2).

### Theme 3: family, peer and social expectations

Family, peer, and social expectations also emerged as an important theme influencing pharmacy students’ wellbeing across both cohorts shaping their sense of responsibility, self-worth, and perceived academic and professional success. Pakistani students reported comparatively negligible family responsibilities during their studies. Several Pakistani students reported their families often adopted a protective or supportive role that prioritised academic achievement, further reinforcing this pattern (Table 3). In contrast, Australian pharmacy students more frequently described significant family responsibilities, including caregiving roles, financial contributions, and house maintenance responsibilities (Table 2). Some Australian students further highlighted peer pressure and peer comparison as prominent contributors towards stress, which added to their overall burden. *“Friends kind of see me as ‘the smart one’ which adds peer pressure about how I am perceived if I fail.”* (A5).

### Theme 4: culture, stigma and attitudes towards mental health

Culture, stigma, and attitudes toward mental health emerged as a critical theme among Pakistani pharmacy students. Participants reported stigmatism and psychological distress was often interpreted through religious or spiritual lenses such as a test of faith or a matter to be addressed through increased religiosity rather than clinical support. A participant quoted, *“Many people are not informed. Some frame it mainly in religious terms like, pray more and overlook medical aspects. Stigma exists.”* (P4). Culture, stigma, and attitudes toward mental health also emerged among Australian participants although stigma was primarily attributed to older generations rather than peers. Participants noted that mental health difficulties were more openly discussed amongst younger people. One participant noted that, although mental health is frequently described as important, this recognition is not consistently reflected in attitudes or behaviours (Table 2).

### Theme 5: awareness and knowledge

Awareness and knowledge emerged as constrained among Pakistani pharmacy students, with participants highlighting limited access to reliable information and a lack of relevant research on mental health. A participant stated, *“There is inadequate amount of information and research regarding mental health in Pakistan…. There is no real concept of mental health and wellbeing in our education system….”* (P1). For their personal awareness of witnessing mental health difficulties, several Pakistani participants described this experience, as reflected in the quotations presented in Table 3. Australian participants reported comparatively greater awareness and knowledge of mental health, supported by public discourse, university initiatives, and exposure to mental health education. Five Australian participants indicated that they had either completed or were currently enrolled in a Mental Health First Aid (MHFA) training program. Programs like this are not available in Pakistan.

### Theme 6: coping strategies and support mechanisms

Coping strategies and support mechanisms varied across participants and contexts. Participants described relying on a combination of individual strategies such as self and creative coping, lifestyle-based coping, and self-management strategies to manage stress and emotional challenges. Among Pakistani pharmacy students, informal support from friends, family and in some cases, teachers were commonly utilised, particularly when professional help was perceived as inaccessible or stigmatised. In contrast, Australian pharmacy students were more willing to seek professional help. When discussing preferred sources of support, one participant stated, *“Psychological help—seeing a counsellor and psychologist”* (A2). For some participants, it depends on the type of issue and situation, with help-seeking varying with the nature and severity of the concern (Table 2). Participants in both countries described diverse self-initiated coping strategies to manage stress such as maintaining social connections (friends and family), taking intentional breaks or using distraction to reset, engaging in creative and hands-on activities (e.g., writing, reading, hobbies, music), practising faith-based coping, using structured breathing techniques, and incorporating physical activity to restore emotional balance.

### Theme 7: help-seeking behaviour and barriers

Pakistani participants reported barriers to help-seeking such as access, awareness, financial constraints, and stigma. A participant expressed, *“In our culture, people do not understand and might judge you if you see a doctor or counsellor…... Financial barriers also play a role”* (P1). These barriers often limited students’ willingness or ability to seek appropriate support. With Australian participants, help-seeking depended more on their individual situation with decisions influenced by the type of issue, perceived need, and personal preferences (Table 2).

### Theme 8: institutional and system-level support

Institutional and system-level support emerged as a critical factor shaping students’ mental health experiences. Participants discussed the role of universities, professional bodies, and healthcare systems in providing accessible services, clear referral pathways, and supportive academic environments. Australian participants acknowledged the availability of counselling services and wellbeing initiatives with some perceiving these supports to be fragmented, difficult to navigate, or insufficiently responsive to students’ needs (Table 2). However, Pakistani participants reported there were no institutional support systems for students seeking help for mental health issues (Table 3). This organisational shortcoming was consistently reported across interviews, a participant reflected, *“Not institutionally—no formal mental-health service. A couple of teachers are open to discussion informally”* (P5).

## Discussion

This qualitative study identified eight shared overarching themes in the accounts of pharmacy students from Australia and Pakistan, but the experiences represented within these themes were not identical. Although academic pressures were common among students in both countries, differences emerged in their educational experiences, sociocultural perceptions of mental health, and access to institutional support services. Taken together, the findings suggest that help-seeking is shaped not simply by whether students recognise a mental health concern but also by whether support is perceived as acceptable, accessible, and responsive within their educational and social environments.

### Educational factors

Academic workload, repeated assessments, and the demands of pharmacy training featured prominently in both cohorts. These findings align with pharmacy education research documenting the relationship between time demands, academic stress and student wellbeing [10,37], however, participants highlighted different aspects of their assessment experiences. Australian students described anxiety associated with objective structured clinical examinations (OSCEs) whereas Pakistani students reported uncertainty surrounding viva examinations (oral assessments in which students respond to questions posed by examiners), particularly when assessed by unfamiliar external examiners. Although anxiety surrounding OSCEs has been investigated in pharmacy education [38], the present interviews add a comparative perspective by identifying uncertainty about assessment expectations as a concern across distinct examination formats. The finding is interpretive rather than a direct evaluation of assessment systems. These accounts do not establish that one assessment method is inherently more stressful than another. Rather, they suggest that assessment preparation and transparent communication of expectations may be relevant alongside workload management.

In addition to these assessment-related concerns, some Pakistani participants reflected on disruptions to earlier educational experiences during the COVID-19 pandemic, particularly through interruptions to teaching and challenges adjusting to university study. Pandemic-era research provides context for these accounts [39], although the interviews cannot establish whether such effects persisted at the time of data collection.

Importantly, educational pressure did not occur in isolation from students’ lives outside university. Australian participants more often described managing employment and household or relationship commitments alongside study whereas Pakistani participants commonly described practical family support for education accompanied by expectations of academic success. Research on pharmacy students’ time use and wellbeing, together with research on family and academic stress, supports examining these pressures together [10,40,41]. A qualitative study of medical students likewise described how academic demands intersected with employment, relationships, and opportunities for recovery [42]. In the present study, however, differences in employment, living arrangements, or year of study could also have contributed to these patterns. The educational implication is, therefore, not that one national curriculum or student group is intrinsically more demanding but that academic support may need to account for the distinct responsibilities and assessment-related concerns students describe.

Participants also described how academic pressures affected their personal wellbeing, including emotional strain and difficulties balancing study with other aspects of their lives. Similar pattern has been reported in pharmacy education research, where heavy workloads and time pressure have been associated with emotional distress, poorer wellbeing, and reduced work–life balance [37,43]. Although the interviews cannot establish the severity or clinical significance of the distress described, these findings highlight the importance of considering students’ emotional experiences alongside academic demands. Mental health concerns, including burnout, anxiety, and occupational depression, have also been reported among healthcare professionals in Pakistan [44]. These findings highlight the importance of supporting pharmacy students in developing appropriate coping strategies and help-seeking behaviours that may be valuable during their education and future professional practice.

### Sociocultural factors

Family and peer relationships were important in both cohorts, although they could operate as sources of reassurance and pressure simultaneously. Pakistani participants often described families as invested in educational success and as trusted sources of emotional or practical support; some also experienced expectations surrounding achievement. Australian participants discussed family and household responsibilities, peer comparison, and the challenge of maintaining social relationships alongside academic demands. These accounts indicate that the influence of social relationships cannot be categorised simply as protective or stressful.

Participants’ accounts also suggested differences in the perceived social acceptability of discussing mental health. Among Pakistani participants, concerns about mental health stigma and social judgement suggest that broader sociocultural beliefs may influence how mental health difficulties are understood and whether seeking professional support is perceived as appropriate. Pakistani participants also described religious coping strategies, including prayer and Quran recitation, as ways of managing psychological distress. Previous research in South Asian contexts has similarly highlighted the influence of stigma, sociocultural beliefs, and religious practices on perceptions of mental illness and mental health help-seeking [45]. Australian participants, in contrast, perceived greater openness towards mental health discussions among peers and reduced stigma among younger people compared with older generations. Earlier systematic review evidence has identified stigma, embarrassment, and concerns about negative social judgement as prominent barriers to mental health help-seeking among young people [17]. Although these findings may suggest changing perceptions of mental health among younger Australians, the present study cannot establish broader generational shifts.

In both cohorts, informal coping strategies and support from trusted individuals were important aspects of students’ responses to psychological distress. Although family and friends may encourage professional help-seeking, reliance on informal support may also reflect barriers to formal mental health services, including limited-service availability, stigma, and concerns about confidentiality. Previous research has identified stigma, confidentiality concerns, and preferences for self-reliance as barriers to professional mental health help-seeking among young people [17]. Importantly, coping strategies should not be conflated with professional help-seeking, as students may manage psychological distress through personal strategies or informal support without necessarily considering professional help. Future research could explore how informal support networks influence pharmacy students’ decisions to seek professional help.

### Institutional and system-level factors

The most consequential contrast in participants’ accounts between the two samples concerned their experiences of formal support within university. Australian students generally recognised that counselling or wellbeing services existed although some described difficulties with waiting times, accessibility, or responsiveness. Pakistani participants more frequently described absence of formal provision in their own institutions, with friends, relatives, and approachable teachers consequently becoming important sources of help. These findings highlight the importance of distinguishing between the availability of mental health services, students’ awareness and accessibility of these services, and the extent to which available support meets their needs. Previous research among pharmacy students in the United States identified lower utilisation of campus counselling services and less awareness of available support compared with medical students [8]. These findings further highlight the importance of ensuring that pharmacy students are aware of available mental health services and are supported in accessing them when needed.

Beyond service availability, participants’ accounts suggest that practical and individual barriers may further influence engagement with professional mental health support. Mental health literacy emerged as another relevant consideration, with Australian participants more frequently describing exposure to mental health education, whereas Pakistani participants reported comparatively limited information and awareness. A systematic review of mental health literacy in Pakistan similarly identified gaps in mental health knowledge and awareness, highlighting the need for improved mental health education [46]. However, recognising mental health difficulties does not necessarily translate into professional help-seeking, particularly when practical or institutional barriers remain. A multicentre study of pharmacy students in the United States identified time constraints, financial limitations, and preferences for self-management as barriers to accessing mental health care [47]. Together, these findings highlight the importance of addressing both mental health awareness and practical barriers when developing support strategies for pharmacy students.

Collectively, the educational, sociocultural, and institutional factors identified in this study suggest that effective mental health support requires more than encouraging students to seek professional help. Students need to recognise when support may be required, understand the available options, and perceive these services as accessible, confidential, and appropriate to their needs. Conversely, expanding services without addressing stigma, confidentiality concerns, time constraints, or perceived need may not ensure their use [8,17,47]. Accordingly, institutional strategies should consider academic pressures, mental health literacy, informal support networks, and clear referral pathways alongside the availability of professional mental health services.

## Strengths and limitations

This study offers a comparative qualitative account of pharmacy students’ mental health and help-seeking across two educational and sociocultural settings. Recruitment across multiple pharmacy institutions in Australia and Pakistan provided perspectives from students studying in different educational and geographical contexts. In Australia, shared accreditation requirements provide some consistency in pharmacy education standards across institutions, including expectations relating to student wellbeing. Pakistan also has national standards for pharmacy education, although student mental health and wellbeing are not addressed in pharmacy accreditation standards. Semi-structured interviews enabled discussion of sensitive experiences in participants’ own terms. Examining country-specific accounts before developing the cross-country comparison helped retain differences in subthemes within eight shared overarching themes. These features strengthen the contextual depth of the analysis. Additionally, this study contributes to the existing literature by comparing pharmacy students’ mental health experiences between a high-income country (HIC) and a lower-middle-income country (LMIC), highlighting contextual differences in how students experience and manage mental health challenges.

This study has several limitations. Although participants were recruited from multiple universities in both countries, the final sample comprised eight students from five Australian institutions and ten students from seven Pakistani institutions. The small sample size, convenience sampling, and uneven participation across institutions may have limited the range and diversity of experiences captured. Therefore, the findings should be interpreted with caution and as context-specific accounts rather than representative of all pharmacy students or institutions in either country. The timing of the interviews should also be considered when interpreting the findings, particularly participants’ reflections on experiences during the COVID-19 pandemic. As pharmacy education in Pakistan typically spans five years, some participants may have experienced pandemic-related educational disruptions during their earlier years of study, potentially influencing their retrospective accounts of academic challenges. Additionally, participants were enrolled in different years of their pharmacy programmes, which may have influenced their experiences of academic stress and help-seeking behaviours. For instance, students in earlier years may experience stress associated with transitioning to university, whereas those in later years may face greater clinical and assessment demands, alongside pressures related to graduation and future employment. Participants who volunteered may also have been more willing to discuss the topic of mental health, which could influence the perspectives captured.

## Implications for research and practice

For pharmacy educators, the shared accounts of academic pressure highlight the need to consider the cumulative burden of assessments and workload while recognising differences in examination-related concerns and responsibilities outside university. Potential approaches include clearer communication of assessment expectations, opportunities for students to discuss academic difficulties, and stronger connections between academic guidance and wellbeing support. However, the effectiveness of these approaches in reducing psychological distress requires further evaluation.

The findings also suggest different priorities for institutional mental health support across the settings represented. Where counselling and wellbeing services are established, universities should consider improving students' awareness of available support and addressing concerns about accessibility, confidentiality, timeliness, and responsiveness. Where participants reported limited formal support, universities could explore feasible and confidential referral pathways alongside mental health literacy initiatives. Trusted academic staff and peers may help students identify appropriate services although responsibility for professional mental health care should not be placed solely on families, peers, or teaching staff.

Future research involving a broader range of pharmacy institutions and student populations could examine how service availability, trust, and accessibility influence help-seeking decisions. Longitudinal studies may also provide insight into changes in academic stress, coping strategies, and help-seeking behaviours throughout pharmacy training. Furthermore, co-designed mental health support strategies should be evaluated for their acceptability, feasibility, and effectiveness, particularly in resource-constrained educational settings. Overall, the findings highlight the importance of addressing educational pressures, sociocultural influences, and institutional opportunities for help-seeking when supporting pharmacy students' mental health. While the shared themes reflect common challenges across both countries, differences in students' experiences emphasise the need for context-sensitive approaches to mental health support.

## Conclusion

This study demonstrates that pharmacy students in Australia and Pakistan experience significant mental health challenges, although the sources of stress, available supports, and help-seeking patterns differ across contexts. While academic pressure was common in both settings, cultural norms, institutional structures, and awareness levels shaped how students understood and managed their wellbeing. These findings highlight the importance of context-sensitive approaches to student mental health within pharmacy education. Strengthening institutional support and promoting open discussion around mental health remain important steps in supporting the wellbeing of future pharmacists.

## Supplementary Material

Supplementary_File_S4 clean.docx Supplementary_File_S4 clean.docx

Supplementary_File_S3 clean final.docx Supplementary_File_S3 clean final.docx

Supplementary File S2.docx Supplementary File S2.docx

Supplementary_File_S1 clean.docx

## Data Availability

The authors confirm that the data supporting the findings of this study are available within the article and its supplementary materials.
