# Supplementary material for: Contextual influences on mental health in pharmacy education: a comparative study across higher- and lower-middle-income settings
Source: Med Educ Online. 2026 Nov 5;31(1):2756743. doi: 10.1080/10872981.2026.2756743 (PMC13757898; doi:10.1080/10872981.2026.2756743)
Supplement: Supplementary file 1 [file ZMEO_A_2756743_SM0015.docx]

**Supplementary File S4:**

1. **Coding numbers and subthemes for Pakistani participants**

| **Code numbers** | **Subthemes** |
| --- | --- |
| **Academic Stressors** | |
| 1-5 | Workload and Time Pressure |
| 6-13 | Exams and Assessments |
| 14-21 | External Disruptions and Personal Consequences |
| **Personal Experiences and Well-being** | |
| 22-23 | Health and Functioning |
| 24-26 | Self-Reflection and Emotional Well-being |
| **Family, Peer and Social Expectations** | |
| 27-30 | Family Pressures and Responsibilities |
| 31-33 | Peer Pressure and Competition |
| 34-37 | Financial and Structural Barriers |
| **Culture, Stigma and Attitudes to Mental Health** | |
| 38-39 | Stigma and Judgement |
| 40-41 | Comfort Levels |
| 42-46 | Barriers and Variation in Attitudes |
| **Awareness and Knowledge** | |
| 47-48 | Awareness Gaps |
| 49-50 | Role of Education |
| 51-52 | Personal Awareness and Reflection |
| **Coping Strategies and Support Mechanisms** | |
| 53-57 | Family Support Systems |
| 58-61 | Peer and Social Support |
| 62-64 | Professional Help |
| 65-75 | Self and Creative Coping |
| **Help-Seeking Behaviour and Barriers** | |
| 76-77 | Financial and structured barrier |
| 78 | Stigma and Judgement |
| **Institutional and System-Level Support** | |
| 79-84 | Systemic Barriers |
| 85-88 | University Environment |
| 89-91 | Counselling Infrastructure |
| 92-99 | Curriculum and Assessment Design |

2. **Codes and their representative quotations From Pakistani Participants**

| **Codes** | **Representative Quotations** |
| --- | --- |
| 2. Time Management Issues | “In 2nd semester a couple of subjects were tough, but I caught up and it's been smooth ever since. Time management is a real issue which is really challenging for me to control right now” (P7).  “I try to keep a schedule. Sometimes circumstances make it hard. After mid-terms I often quit any part-time commitments and go full-time on studies. It’s a constant adjust-and-replace approach” (P2).  “Time constraints—around exams the syllabus is intense and time feels insufficient” (P7). |
| 3. Heavy Workload | “Heavy syllabus, so many subjects to cover in a short time, and draining lab work causes difficulty in managing time” (P8). |
| 4. Workload Management Strategies | “I also help with my father’s chores and manage a long commute. On heavy weeks—quizzes, preparation, early mornings—the workload goes up a lot. A couple of days ago I was revising for a quiz while unwell (cold/fever). Balancing everything gets tough” (P2). |
| 5. Impact of Workload on Personal Time | “There are many family events I can’t attend because semesters move quickly: quizzes, papers, then results and remedials. I have to give time to studies, so attendance at family functions is low” (P4).  Yes—especially around 5th to 9th semesters. The load was heavy. Final-year project research alongside regular courses was very demanding; even managing family time became difficult” (P6).  “Yes—social life shrinks. I reach home late, so there’s little time for friends or even family on weekdays” (P7). |
| 6. Not Affected by Workload | “I try to do different things to relieve myself, so the workload hasn’t affected me too negatively” (A1). |
| 7. Assessment Anxiety | Yes—the university suddenly shifted final assessments to online objective exams after years of on-campus written papers……. That abrupt change was mentally disturbing for many of us" (P5). |
| 8. Grade Anxiety | “Early on I feared grades—more precisely, others’ reactions specifically family and friends to my grades…...” (P9). |
| 9. Reduce Grade Obsession | “Reduce grade obsession and increase faculty support….” (P10). |
| 10. Unclear Exam Process | “In vivas you do not know what will be asked, external examiner is not much familiar with what core topics have been taught, so this uncertainty creates stress” (P5). |
| 11. Academic success prioritised over mental health | “I give more preference to academics. I adjust the ratio—some days 60/40, other days 75/25—depending on what’s due” (P3).  “I use free time to finish academic tasks first—assignments, prep, etc. Earlier I went out more; now I stay home more and focus on studies” (P7). |
| 12. Subject Difficulty | “In 2nd semester a couple of subjects were tough, but I caught up and it's been smooth ever since” (P7).  “Physiology was especially hard; our MBBS doctor taught beyond our syllabus………” (P10). |
| 13. Theory-Practice Gap | “A functional gap between what we study and what industry does. We study Industrial Pharmacy, but in five years we got only one industry visit. When I visited, the setup felt very different from our books. That lack of real exposure has been a challenge” (P5). |
| 14. Academic Decline | “From the very first year, COVID hit…... I could not do well in studies because I was not familiar with digital platforms and equipment” (P1). |
| 15. Covid-19 Disruptions | “Starting university in 2020 during COVID—everything was online. As a first-semester student, there was no proper guidance, lectures were hard to follow, and my GPA suffered” (P6). |
| 16. Studying under pressure is a choice | “I tend to study only under pressure” (P4). |
| 17. Performance Pressure | “Transitioning from FSc to undergrad brought a big pressure shift. You feel you must prove yourself—to parents, teachers, and yourself” (P9). |
| 18. Balancing Academics & Life | “It’s hard. You need patience and tolerance—patience is key. I try to keep steady despite competing demands” (P6).  “Early on I leaned toward academics; now I’m at a better place—able to balance both” (P8). |
| 19. Stress Impacting Mood | “Yes—stress is primary; if it becomes overwhelming, anxiety/low mood follows” (P8). |
| 20. Emotional Struggle | “Family supported me, but I felt low and somewhat depressed” (P6). |
| 21. Lack of Routine | “I tend to study only under pressure. On ordinary days I don’t keep a routine; when exams/quizzes approach, the pressure makes me study. Without that pressure, it’s hard to sit and read” (P4). |
| 22. Future Anxiety | “Thinking about jobs and the future makes me question my credibility, even though I was once a top student” (P1). |
| 23. Physical health negative impacts | “I do struggle with daily headaches. It happens almost every day due to long study hours, and it affects me a lot” (P1). |
| 24. Motivation and Productivity | “Keep yourself motivated. Prepare the curriculum regularly and use weekends to clear backlogs. Replace social media scrolling with productive hobbies or passions. Spend time on what energises you; it helps mental health” (P2). |
| 25. Self-Awareness | “The key is self-awareness. If your environment is causing the problem, change it if you can—growth is hard in the wrong environment. If you can’t change it, build supportive people around you and take care of basics like diet and routine” (P3). |
| 26. Detaching sense of self from academic performance | “….Learning to accept a “B” and detach my worth from marks reduced that fear” (A9). |
| 27. Emotional Struggle | “Due to emotional drain, I felt low and somewhat depressed. Then I worked hard, improved each term and recovered” (P6). |
| 28. Family Responsibilities | “No special responsibilities—family wants me to focus on studies” (P7). |
| 29. Limited Family Time | “Mainly limited time with family on weekdays; most talking happens on weekends” (P7). |
| 30. Household Responsibilities | “No formal financial responsibility, but I help with errands and day-to-day tasks at home” (P2).  “As an elder/household lead when needed, I look after siblings and handle documents/errands (especially when my father is away)” (P4). |
| 31. Adjusting Commitments | “After mid-terms I often quit any part-time commitments and go full-time on studies. It’s a constant adjust-and-replace approach” (P2). |
| 32. Peer Pressure | “…..Peer pressure was real—my first mid-term marks were low, and I doubted if I could continue” (P10). |
| 33. Peer Competition | “…. Seeing peers excel in community work, sports, research, made me fear “Will I graduate and still be unknown here?” The anxiety of that troubles me” (P9). |
| 34. Identity Struggles | “………. Building an identity on campus was stressful….” (P9). |
| 35. Travel burden | “In the first 2–3 years it was tough. I had weekly four-hour one-way travel (Islamabad), sometimes missing classes due to home tasks” (P5). |
| 36. Financial Barriers | ““Honestly, I usually don’t bother seeking help. In our culture, people don’t understand and might judge you if you see a doctor or counsellor. There are services available, but students don’t feel comfortable using them. Financial barriers also play a role” (P1). |
| 37. Financial Constraints | “Yes—seeing peers excel in community work, sports, research, made me fear “Will I graduate and still be unknown here?” The anxiety of that troubles me. Academically I managed, but personal financial crises created imbalance. I sometimes felt anxious and isolated. Over time I learned to control my anxiety” (P9). |
| 38. Limited Resources/Access | “Limited resources and access are major barriers” (P6).  “First, talk to family; then to friends; and consult a professional if possible. In Pakistan, access is limited and finances can be a barrier—but counseling helps. Also, some people take unnecessary tension—help them reframe and manage it” (P7). |
| 39. Stigma | “Fear of family/friends finding out and attaching a label; social stigma. Finances can also be a barrier” (P5).  “There is stigma and worry about what others will think. I usually go to friends; a concern is whether they’ll have time or be emotionally available” (P3). |
| 40. Fear of Judgement | “What will people say?” Social pressure. If someone seeks help, they may hide it for fear of being labeled “crazy.” That’s a barrier” (P4).  “There’s stigma if you disclose mental health problems, people may label you “crazy,” which can affect your future. So most prefer to hide it—even from family” (P5).  “Stigma—people’s judgments. You have to be strong; if you keep listening to what people say, you can’t move forward. Psychosis and related issues are increasing, yet treatment access is limited; Pakistan ranks high in prevalence, and addressing it is difficult” (P6).  “It could be taken negatively or made fun of—that’s a barrier. Acceptance is low” (P7).  “Fear of being labeled “mental/mad.” A trusted teacher advised keeping my therapy private while I was emotionally fragile; I did so to avoid negative reactions” (P10). |
| 41. Not Comfortable Discussing | “I am not comfortable. In our society, people judge you if you share personal struggles, especially about mental health. So, I prefer not to discuss it” (P1).  “No, most are still confined and hesitant. The stigma is breaking slowly, but there’s still a long way to go” (P1).  “No” (P2).  “No” (P3). |
| 42. Comfortable Discussing | “It depends. With friends who understand, I’m comfortable. With those who don’t, it’s hard—they may dismiss or blame” (P4).  “Only with one close friend. I don’t share with the class or family; people can bring it up later in groups and make it awkward” (P5).  “With trusted friends I’m very comfortable; they can counsel well” (P6).  “Very comfortable. I talk openly and explain things; my friends share with me too” (P7).  “Comfortable with close friends and parents; others often don’t take it seriously” (P8). |
| 43. Mixed Attitude | “Mixed—some take mental health seriously, some minimise it” (P3).  “Mixed. Some treat it as a medical issue that needs management; others view it primarily through a religious lens and don’t accept it as a health condition” (P4). |
| 44. Girls More Comfortable | “Among my class, girls tend to be more comfortable discussing mental health than boys, though it varies by person. You can’t fully generalise” (P9). |
| 45. Positive Peer Support | “Yes. I’ve seen classmates who appear to struggle; many seek teacher counseling. Hostelites especially face loneliness and depression—being away from home. Classmates and friends often support them, which is positive” (P6).  “Yes. I observe well—changes in behavior tell me. If it’s a close friend, I confirm and talk it through” (P7).  “In Pakistan, there is almost no concept of mental health. It’s slowly being established, and people are beginning to take it seriously, but there’s still a long way to go. I’m also trying to contribute—we’ve started a group with the help of a friend who supports me in this” (P1).  “My close friends believe we should talk, support, and counsel one another so everyone can progress” (P6).  “My close friends are like me—open and willing to share and discuss” (P7). |
| 46. Low Acceptance | “It could be taken negatively or made fun of—that’s a barrier. Acceptance is low” (P7).  “Many frame issues spiritually (e.g., “possession”) rather than medically, so psychology/psychiatry isn’t the first step—this creates delay and stigma” (P8). |
| 47. Mental Health as Low Priority | “It isn’t prioritised. In clinics, physical issues are checked, but underlying depression/anxiety or causes behind illness get little attention. There’s stigma: if you disclose mental health problems, people may label you “crazy,” which can affect your future. So most prefer to hide it—even from family” (P5).  “Poorly—especially by older generations. People may label someone “mental” or “mad” instead of offering support. Counseling culture is weak; even siblings/families may not counsel well. Good friends can help, but overall there’s a negative cultural stance” (P6).  “There are many mental-health issues around—friends get stressed, anxious, sleep-deprived near exams—but society doesn’t value mental health enough. It’s not focused on much” (P7). |
| 48. Lack of Awareness | “In Pakistan, there is almost no concept of mental health. It’s slowly being established, and people are beginning to take it seriously, but there’s still a long way to go. I’m also trying to contribute—we’ve started a group with the help of a friend who supports me in this” (P1).  “There’s no real concept of mental health and wellbeing in our education system, it needs reform. Specifically in pharmacy, we should have courses teaching students how to maintain mental health and wellbeing” (P1).  “A psychologist is reportedly available, but awareness is low—more communication and visibility are needed” (P8).  “There is awareness, but it’s uneven. People know terms like “depression” or “anxiety,” yet understanding of what they really mean and how to respond is limited” (P3). |
| 49. Limited Information | “No; I don’t recall such sessions being offered” (P3).  “No—no sessions I’ve joined, and opportunities/interest haven’t aligned” (P4).  “No—opportunities are scarce, and universities rarely open this topic” (P6).  “No—never seen such trainings around me” (P7). |
| 50. Education Increasing Awareness | “We need much more work—awareness programs, workshops, conferences—so people realise mental health matters. If universities and teachers lead, it can help students, and eventually even older generations will understand once they’re made aware” (P6).  “Universities in Pakistan need much more awareness work—students face anxiety/depression but rarely talk about it. Greater visibility and supportive services could make a real difference” (P8). |
| 51. Medical Students More Aware | “Among medical students, there’s more understanding, so I can discuss it. With local/non-medical friends, I usually don’t” (P2).  “Despite being medical students (slightly more aware), many still mirror family/cultural views—limited openness” (P8). |
| 52. Valuable Research Topic | “This is a very good study. Research like this should be practised in Pakistan. We don’t have a strong research culture, and even my own research lacks supervision. Studies like yours are needed” (P1).  “Your research on students’ mental health is a good initiative” (P4). |
| 53. Personal Awareness of Others | “I think I’m more aware. I try to observe others—how they perform in class, their marks, their behaviour. That gives me some sense of their wellbeing. But overall, I don’t think my peers are very aware” (P1).  “I notice demotivation—poor mid-term results, perceived favoritism, classroom environment. I encourage friends to stay strong and focus on projects, presentations, assignments—keep going” (P2).  “Often, yes—like when someone isolates suddenly. If someone is very withdrawn, I sometimes hold back, but generally I can sense it” (P3).  “I think I am aware. One pharmacy friend has panic attacks, especially around exams, and attends counselling. Early on people misread it as lack of religiosity; later they understood it’s medical. Many students show higher anxiety near exams” (P4)  “I notice when friends are withdrawn or low. Common triggers are finances, academic pressure, and relationships. I try to check in” (P5).  “Yes. I’ve seen classmates who appear to struggle; many seek teacher counseling. Hostelites especially face loneliness and depression—being away from home” (P6). |
| 54. Family as First Line of Support | “I believe you should take help. First, I’d discuss with family, then friends. They’re closest and understand me; I’d take their advice on next steps” (P7).  “The biggest factor is how open I can be with my parents. If my parents back me, society can’t derail me. They’d first try to counsel me; if it’s beyond them, they’d support professional help” (P9).  “Family and friends first (family before friends). Family can support me better I guess because there is no one who cares more for me than my family” (P7)  “Parents first; then friends/teachers for advice and a counselor referral if needed” (P8).  “Depends on the issue. For academic problems—teachers. For personal matters—parents first then friends. If it’s beyond all of us, then a professional counselor/psychiatrist” (P9). |
| 55. Family Expectations | “During exam periods family expectations rise and I feel closely “tracked.” Time pressure plus that scrutiny is stressful” (P3).  “My brother is very religious and expects me to go down a certain path, but I’ve always felt I’m not made for that. I try my best, but sometimes it creates pressure. My family expects a lot from me. As for peers, I don’t feel much social pressure” (P1).  “Families here have a traditional mindset—doctor or a basic salaried job. Alongside pharmacy I’m a music producer. I’m working on collaborations (including some paid projects) with local and international artists. Ideally, the degree will become the “part-time” and music the “full-time.” Family pressure exists, but I self-motivate” (P2).  “Family wants “doctor-level” outcomes. My brothers are doing well (judiciary), so expectations are high. People often don’t distinguish pharmacist vs physician. I try to meet expectations by strengthening my clinical knowledge and explaining our role. Initially it stressed me; now I can set boundaries and clarify what pharmacists do” (P5).  “Families do expect you to do better and better. If you don’t meet expectations, they’re disappointed—and that affects your mental health. I try to keep a positive mindset: work hard, aim to meet expectations, but also know your limits and care for yourself” (P6) |
| 56. Supportive Family | “Not much. If I achieve, they’re happy; if I fall short but have worked hard, they understand. So no major negative impact” (P4).  “I’m fortunate—no major financial responsibilities. Family supports me emotionally and financially. Personally I do feel at this age I “should be earning,” but they tell me to focus on studies” (P5).  “No special responsibilities—family wants me to focus on studies” (P7).  “No—main focus is to study well” (P8). |
| 57. Positive Expectations | “Yes—in a positive way. Their expectations push me to prepare well and work harder. Before university, we studied without clear goals; now I feel the responsibility” (P7). |
| 58. Can Hide if Needed | “Personally, I wouldn’t face big challenges. I’ve been exposed to different environments, so I could even hide it from my family if needed. But yes, in our society, openly seeking help could raise questions and stigma” (P1). |
| 59. Peer Support | “Friends—because they understand my context and may have faced similar issues, so it’s easier to explain and get practical support” (P3).  “First, I will consult close friends for advice; second, a psychologist/counsellor if needed” (P4).  “Trusted close friends. Some teachers are approachable on a personal level, but there’s no formal university support system I’m aware of” (P5).  “Friends first. I feel a friend is same age group to me and can understand me better” (P6). |
| 60. Trusted Friend | “Find one trustworthy friend you can speak to freely (and who won’t share it later). Read helpful books on mental health and coping” (P5). |
| 61. Reliance on Friends | “There is stigma and worry about what others will think. I usually go to friends; a concern is whether they’ll have time or be emotionally available” (P3). |
| 62. External Support | “We need more professional, accessible psychological support. Regular, student-focused mental-health initiatives would help” (P5).  “We need much more work—awareness programs, workshops, conferences—so people realise mental health matters. If universities and teachers lead, it can help students, and eventually even older generations will understand once they’re made aware” (P6).  “Many friends lose sleep and get very tense before papers. Counseling and short workshops could help a lot” (P7). |
| 63. Professional Help | “First, trusted teachers/doctors at the university. There are counsellors and a Psychology department; a counsellor could advise. So, I would begin within the university system” (P2).  “First, I will consult close friends for advice; second, a psychologist/counsellor if needed” (P4).  “Reduce grade obsession and increase faculty support. Appoint a university psychologist who can counsel students and present student concerns to faculty” (P10).  “Seek professional help. Therapy felt taboo at first and I was hesitant to open up, but in later sessions I shared more and it helped. A good counselor can highlight your strengths/weaknesses so you can focus on yourself and tune out the noise” (P10). |
| 64. Counselling if Available | “Unfortunately, students often have to rely on themselves. My advice is to self-reflect and identify what’s bothering you. Try to be your own healer. If support is available, definitely go for counselling, but if it’s not, you have to help yourself” (P1). |
| 65. Underutilised Support System | “There is a system—teachers/doctors are approachable; counselling may be available. It depends on whether students actually approach and use it” (P2). |
| 66. Self-Intervention | “From second semester on, I worked hard, improved each term, and Alhamdulillah recovered—though I still fear disappointing my parents/friends. Faith helped me through” (P6).  “Unfortunately, students often have to rely on themselves. My advice is to self-reflect and identify what’s bothering you. Try to be your own healer” (P1).  “Negative incidents (e.g., theft, setbacks) trigger overthinking and stress. Try to reframe—look for the lesson and the opportunity—so it doesn’t spiral” (P2).  “Mental health is a broad topic; you can’t force neat conclusions. The key is self-awareness. If your environment is causing the problem, change it if you can—growth is hard in the wrong environment. If you can’t change it, build supportive people around you and take care of basics like diet and routine” (P3). |
| 67. Creative Coping (Poetry) | “I write poetry. Whenever I feel anxious or low, I write down my thoughts. That helps me manage my emotions” (P1). |
| 68. Reading Books | “Find one trustworthy friend you can speak to freely (and who won’t share it later). Read helpful books on mental health and coping” (P5).  “Reading—books helped me a lot with personal issues” (P5). |
| 69. Music | “Music. I play guitar/keyboard and spend time in a small recording setup. It’s my passion/hobby and helps me decompress” (P). |
| 70. Journaling | “I write (journaling/speeches/notes) when I’m overwhelmed—it helps the most. Also going out or talking things through with friends” (P3).  “Journaling—writing thoughts and feelings in the moment. If overwhelmed, I lie down and rest; it helps” (P10). |
| 71. Sleeping | “Positive self-talk and faith (“we’ll manage; this will pass”). Sometimes I nap, take a short break, scroll a bit, then return to study” (P4). |
| 72. Faith | “Faith-based coping reciting Quran (Surah Ad-Duha and other verses) calms my anxiety and helps me through tough times” (P6). |
| 73. Walking | “When I get home, I give myself some time—go for a walk/boxing, think through plans, set a timetable, and use even small pockets of time well” (P6). |
| 74. Boxing | “When I get home, I give myself some time—go for a walk/boxing, think through plans, set a timetable, and use even small pockets of time well” (P6). |
| 75. Box Breathing | “Box-breathing (inhale ~4s, hold, exhale ~4s) before stressful tasks; maintain social connections—talk, laugh, go out; keep strong ties with parents” (P8). |
| 76. Limit Use of Social Media | “Keep yourself motivated. Prepare the curriculum regularly and use weekends to clear backlogs. Replace social media scrolling with productive hobbies or passions. Spend time on what energises you; it helps mental health” (P2).  “Cut down social-media time. Don’t over-interpret every situation. If needed, go for counselling—don’t hesitate” (P3). |
| 77. Financial barrier towards help-seeking | “Honestly, I usually don’t bother seeking help. In our culture, people don’t understand and might judge you if you see a doctor or counsellor. There are services available, but students don’t feel comfortable using them. Financial barriers also play a role” (P1).  “Fear of family/friends finding out and attaching a label; social stigma. Finances can also be a barrier” (P5).  “Severity—impact on daily life/studies/behavior; (2) Finances—cost of sessions/therapy” (P8). |
| 78. Experience With Professional Services | “A genuinely good friend can be better than counseling—someone who listens and guides. I had some mental health problems before, for which I did avail professional psychiatrist service. (My counselor even asked me to make a good friend at university, which I struggled to do.) If I had that, I might not have needed counselling” (P10). |
| 79. Stigma in open help-seeking | “Many want stigma to end but still fear being sidelined if they seek help. I encourage communication—talk to trusted people or professionals rather than staying silent” (P10).  “Honestly, I usually don’t bother seeking help. In our culture, people don’t understand and might judge you if you see a doctor or counsellor. There are services available, but students don’t feel comfortable using them. Financial barriers also play a role” (P1).  “Stigma—fear that “society will know I’m taking therapy”—and whether the person I approach will genuinely give time and support” (P3).  “Personally, I wouldn’t face big challenges. I’ve been exposed to different environments, so I could even hide it from my family if needed. But yes, in our society, openly seeking help could raise questions and stigma” (P1). |
| 80. Educational Issues | **“**During exams I get home late and have very little time to revise a big syllabus. There is usually no gap between papers so that’s hard” (P7). |
| 81. Educational Inequalities | “Our education system has issues classroom environment, noise (coming from outside), and sometimes favouritism. Since school/F.Sc. I have felt that “toppers” are prioritised. It took me years to recognise how that affected me. Coming from rural/feudal areas, many students lack platforms and support. Access and opportunity can be difficult” (P2). |
| 82. Educational system issues | “Our education system has issues classroom environment, noise (coming from outside), and sometimes favouritism. Since school/F.Sc. I have felt that “toppers” are prioritised. It took me years to recognise how that affected me. Coming from rural/feudal areas, many students lack platforms and support. Access and opportunity can be difficult” (P2).  “Yes—the university suddenly shifted final assessments to online objective exams after years of on-campus written papers. Topics teachers had said were “excluded” still appeared (auto-selected by the system), so even strong students’ GPAs dropped (mine too). That abrupt change was mentally disturbing for many of us” (P5). |
| 83. Lack of Guidance | “Medicinal Chemistry has been tough. Sometimes the pace or assumptions make you feel your basics aren’t strong, which creates self-doubt, especially when there isn’t enough guided support” (P3). |
| 84. Pharmacy not First Choice | “Well, pharmacy wasn’t my choice initially, so from the start it was difficult. I don’t recall one specific aspect that made me question my choice, but there is something important: although we are studying pharmacy in Pakistan, there aren’t good opportunities to practise what we learn” (P1). |
| 85. Limited Opportunities | “No. Opportunities may exist, but cultural influence and priorities mean we don’t engage much” (P2).  “Well, pharmacy wasn’t my choice initially, so from the start it was difficult. I don’t recall one specific aspect that made me question my choice, but there is something important: although we are studying pharmacy in Pakistan, there aren’t good opportunities to practise what we learn” (P1) |
| 86. University Adjustment | “The shift from college to university was bigger than I expected. University study isn’t something you can “fit in” around other activities— it needs sustained time. Early on I under-allocated time” (P3).  “Transitioning from FSc to undergrad brought a big pressure shift. You feel you must prove yourself—to parents, teachers, and yourself. Building an identity on campus was stressful” (P9). |
| 87. Strict Teaching Style | “Organic Chemistry (1st prof) and Analytical Chemistry (3rd prof)—the instructor’s very strict, old-school style added pressure” (P8). |
| 88. Supportive Teachers | “Not a particular course. Teachers are generally available and supportive. The broader stress was about finding my place and identity, not a single subject” (P9) |
| 89. Teachers’ Expectations | “To be honest, my parents don’t make many demands—they’re happy with what I’m doing. But my teachers have expectations. In the first three years I was a brilliant student, but now in the final year I feel dull. Thinking about jobs and the future makes me question my credibility, even though I was once a top student” (P1). |
| 90. Lack of Counselling services | “There’s no real concept of mental health and wellbeing in our education system, it needs reform. Specifically in pharmacy, we should have courses teaching students how to maintain mental health and wellbeing. Universities should have active counselling centres, but they don’t. Mental health should be part of the curriculum” (P1). |
| 91. Institutional Counselling not Available | “As far as I know, there isn’t a formal support/counselling service available” (P3).  “As far as I know, there isn’t an accessible, active counselling system available to us” (P4).  “Not institutionally—no formal mental-health service. A couple of teachers are open to discussion informally” (P5).  “Not much. Recently one teacher organised activities and speaks with students; a few teachers advise and discuss depression in class. But university-wide systems are lacking—this needs far more work” (P6).  “No formal system at our university” (P7).  “There are no specific designated mental-health services available. But several teachers are approachable and give thoughtful guidance beyond academics, which has helped me socially and academically” (P9).  “Currently, zero. At orientation we were told a psychologist would be appointed; it hasn’t happened yet. I hope it will in the future” (P10). |
| 92. Lack of Institutional Priority | “We are trying to start something, but it’s very difficult. People don’t prioritise it. At the institute, a psychologist is available; however, their services are limited to external patients and are fee-based, rather than being accessible to enrolled students” (P1). |
| 93. Remove Unnecessary Subjects | “Support is limited. Better curricular linking to reduce mental load. For example, when Physiology covers CNS and Pharmacology also covers CNS, aligning the sequence across subjects would build understanding, reduce stress, and lighten the burden. Stronger cross-linking between related topics would help. Organic chemistry should be eliminated from the curriculum because I think it’s of no use in future” (P2).  “Reduce course load/credit burden—the content and credits are heavy and drive stress. Exam formats and presentations vary by teacher; broader structural support would help, but curriculum/exam control sits with departments” (P4).  “Reduce redundancy and better structure sequencing. Some topics repeat across subjects (e.g., dosage forms/dispensing content), while others (e.g., medicinal chemistry structures) were previously skipped but now included for juniors. Streamline syllabus and remove unnecessary repetition” (P6).  “Shorten the degree to 4 years, remove outdated/irrelevant courses (e.g., non-core subjects at this level), and update the syllabus so students enter the workforce sooner” (P8).  “Add career-specific elements: mandatory final-year internships to bridge theory–practice; introduce final-year electives aligned with a chosen path. Highly specialised subjects (e.g., deep analytical chemistry) fit better at master’s/specialisation level rather than brief undergrad exposure” (P9). |
| 94. Streamline Assessment | “More mentoring and spaces to discuss struggles, so students who won’t speak up in class can still talk. Reduce unnecessary or repetitive assessments/processes; streamline where possible” (P3).  “Reduce course load/credit burden—the content and credits are heavy and drive stress. Exam formats and presentations vary by teacher; broader structural support would help, but curriculum/exam control sits with departments” (P4).  “Reduce redundancy and better structure sequencing. Some topics repeat across subjects (e.g., dosage forms/dispensing content), while others (e.g., medicinal chemistry structures) were previously skipped but now included for juniors. Streamline syllabus and remove unnecessary repetition” (P6).  “Improve the examination system and allow better revision time; timing often feels too tight” (P7). |
| 95. Academics should teach subjects where their expertise lies | “Align tracks with student interest. If someone is industrial, they should teach deeper industry content with real visits; if clinical, focus should be more on clinical. Don’t push everyone across domains purely by rote; reduce mismatch between curriculum and actual practice” (P5).  “Add career-specific elements: mandatory final-year internships to bridge theory–practice; introduce final-year electives aligned with a chosen path. Highly specialised subjects (e.g., deep analytical chemistry) fit better at master’s/specialisation level rather than brief undergrad exposure.” (P9). |
| 96. Redesign curriculum to reduce mental stress | “Support is limited. Better curricular linking to reduce mental load. For example, when Physiology covers CNS and Pharmacology also covers CNS, aligning the sequence across subjects would build understanding, reduce stress, and lighten the burden. Stronger cross-linking between related topics would help. Organic chemistry should be eliminated from the curriculum because I think it’s of no use in future” (P2). |
| 97. Curriculum Integration of Mental Health | “There’s no real concept of mental health and wellbeing in our education system, it needs reform. Specifically in pharmacy, we should have courses teaching students how to maintain mental health and wellbeing. Universities should have active counselling centres, but they don’t. Mental health should be part of the curriculum” (P1). |
| 98. Lack of mental health research | “This is a very good study. Research like this should be practised in Pakistan. We don’t have a strong research culture, and even my own research lacks supervision. My topic is about mental health and people’s perceptions, and how to manage it. Studies like yours are needed” (P1). |
| 99. Lack of expert guidance | “This is a very good study. Research like this should be practised in Pakistan. We don’t have a strong research culture, and even my own research lacks supervision. My topic is about mental health and people’s perceptions, and how to manage it. Studies like yours are needed” (P1). |
| 100. Applying Knowledge to Practice | “I go back to my village every two weeks. I’m very interested in clinical/hospital/retail practice, so I review patients’ files/medicines at our counter, check interactions, and guide on proper administration—within my scope” (P5). |
