# Supplementary material for: Contextual influences on mental health in pharmacy education: a comparative study across higher- and lower-middle-income settings
Source: Med Educ Online. 2026 Nov 5;31(1):2756743. doi: 10.1080/10872981.2026.2756743 (PMC13757898; doi:10.1080/10872981.2026.2756743)
Supplement: Supplementary file 2 [file ZMEO_A_2756743_SM0016.docx]

**Supplementary File S3:**

1. **Coding numbers and subthemes for Australian participants**

| **Code numbers** | **Subthemes** |
| --- | --- |
| **Academic Stressors** | |
| 1-22 | Workload, Exams and Assessment Pressure |
| 23-30 | Time Pressure, Clashing Demands, and Work–Study Balance |
| 31-39 | Transition and Academic Adjustment |
| **Personal Experiences and Well-being** | |
| 40-53 | Social Life, Self-Care and Relationships |
| 54-61 | Financial Stress, Work and Structural Constraints |
| 62-70 | Emotional Burden and Psychological Impact |
| **Family, Peer and Social Expectations** | |
| 71-82 | Family Expectations, Pressures and Ambitions |
| 83-92 | Family Responsibilities and Household Roles |
| 93-103 | Peer Relationships, Comparison and Identity |
| **Culture, Stigma and Attitudes to Mental Health** | |
| 104-122 | Cultural Attitudes, Stigma, and Generational differences in attitudes |
| 123-141 | Openness and Comfort Discussing Mental Health |
| **Awareness and Knowledge** | |
| 142-151 | Awareness of Peers’ Mental Health and Proactive Support |
| 152-160 | Training, Programs and Mental Health Literacy |
| **Coping Strategies and Support Mechanisms** | |
| 161-175 | Professional and informal support |
| 176-185 | Self-Directed, Lifestyle and Task-Oriented Coping |
| **Help-Seeking Behaviour and Barriers** | |
| 186-206 | Help-Seeking Threshold, Decision Rules, and Sources of Help |
| 207-213 | Barriers and Negative Experiences of Help-Seeking |
| **Institutional and System-Level Support** | |
| 214-231 | Views on University Support and Desired Improvements |
| 232-236 | Intensity of Pharmacy Course |

2. **Codes and their representative quotations From Australian Participants**

| **Codes** | **Representative Quotations** |
| --- | --- |
| 2. Pressure during OSCEs and exams | “The crunch is when OSCEs, exams, and compounding practical test come. It’s difficult to manage the workload” (A1). |
| 3. Academic pressure | “There is a lot of academic pressure, especially with hurdle assessments—you have to pass or you cannot progress…..” (A2). |
| 4. Pressure to achieve high grades | “……..Also, hospital jobs require top grades, so we are constantly striving for the best marks” (A2). |
| 5. Stressful workload | “Academic challenges, I suppose, are just adapting to how the assessments come around. In school, it was always a very direct build up to exams, but here, already in week two, I was starting to get assignments. It is definitely stressful in that aspect………..” (A3). |
| 6. Self-directed burden | “………..There is also more self-directed learning, so that puts more workload on me to manage my own study” (A3). |
| 7. Extensive assignments | “…….First year, there was a statistics unit with a final assignment, that was daunting—straight out of high school to that………” (A5). |
| 8. Extensive coursework | “I expected university to be a bit easy, but I was not prepared for the overwhelming amount of content…………” (A6). |
| 9. Draining workload | “The material is not necessarily difficult—it is the quantity. Also, multiple 3-hour practicals for each course take a lot of time, and then there is more work at home. All this workload is draining” (A6). |
| 10. Long study hours | “During assessment blocks I am studying eight to ten hours a day” (A1). |
| 11. Intense study week | “When that kind of week hits, I basically sit in my room studying almost 24/7” (A5). |
| 12. Heavy daily study time | “I am a full-time student, so I allocate time to it every day—about six to eight hours between classes, tutorial preparation, and mandatory learning/certifications” (A8). |
| 13. Study dominates daily life | “Definitely. I can only work one shift a week. I do not remember the last time I went out with friends. It is basically uni all day, every day; if not uni, I am working or sleeping” (A6). |
| 14. OSCE stress | “OSCE’s time is the most stressful time” (A7).  “OSCE’S are designed in a way which creates pressure on students” (A8). |
| 15. Exam stress | “Exam period is difficult” (A2). |
| 16. First year stress | “I cannot say much as it is my first year, but it has definitely caused stress—especially with the early introduction of assignments. It just piled up quickly right from the start” (A3). |
| 17. Assignment pressure | “I cannot say much as it is my first year, but it has definitely caused stress—especially with the early introduction of assignments. It just piled up quickly right from the start” (A3). |
| 18. Normal stress | “I do not believe yet—at least not significantly. There has been healthy, normal amounts of stress and some anxiety, plus learning different uni systems. I would not say it has greatly impacted me—still early days—but there is normal stress” (A4). |
| 19. OSCE fear of failure | “I have had OSCEs where I worried I might fail a unit. In a second-year, second-semester unit I needed around 60% on the OSCE to pass—I was freaking out for ages and looking up what happens if you fail. I ended up doing better than expected. It makes you worry, but I am generally pretty calm” (A5). |
| 20. Exam anxiety | “Chemistry. I did not do it in Year 12, so I am new to it. There is a lot of chemistry in pharmacy, so I have been relearning basics and applying them quickly to more complex topics. It has meant more effort than my peers and more exam anxiety about whether I am up to the standard” (A4). |
| 21. Trimester workload pressure | “This time last year, after moving to Townsville, I missed my family and felt alone. The shift to trimesters increased workload. I also work in two pharmacies, so juggling everything was really difficult” (A7). |
| 22. Content mentally demanding | “I feel pressure to pass because my family helps where they can. I have been told I cannot fail a subject; that would mean an extra year of fees and an extra year of mandatory placement with no income. Pharmacy content is challenging and mentally demanding” (A8). |
| 23. Pharmacy is hard | “Honestly—do not do pharmacy. It is too hard, and I regret it” (A2).  “Pharmacy is very hard and the stress is not discussed enough. Many are overwhelmed and mental health is affected; I wish there were more ways to get help specific to our degree” (A6). |
| 24. Time constraint | “Everything we have learned was only briefly touched on before, and now, in first year, it is “you need to know all this content before placement.” There is a strict cutoff on whether you are ready—yes or no—and there is not really an extension. So the time constraint of taking in completely new information is the big challenge” (A4).  **“**Time management—trying to organise myself and understanding what needs to be done sooner rather than later” (A3).  “Time and cost. As a university student I am pressed for time, and costs are not always covered—so those are the big ones” (A5). |
| 25. Clashing deadlines | “Two things come to mind. First year, first semester, there was a statistics unit with a final assignment. Mine ended up being about 56 pages; I heard some students were around 70. That was daunting—straight out of high school to that. Another time: third year, first semester, I came back from placement—I had an exam on Wednesday, an assignment due Friday, I was working the weekend, and then an exam on Monday” (A5). |
| 26. Work-study conflict | “The main challenge is financial. I come from a low- to medium-income household, and my parents cannot help with study fees, including mandatory placements. Because of that, I have had to work at least part-time for many hours across my degree, which affects my ability to study and adds stress from managing multiple things at once. Academically I feel well supported; it is the time that is limited due to finances” (A8). |
| 27. Limited work hours | “Definitely. I can only work one shift a week. I do not remember the last time I went out with friends. It is basically uni all day, every day; if not uni, I am working or sleeping” (A6). |
| 28. Reduced ability to work | “I do not get Centrelink or scholarships, so I have to support myself. Workload affects the hours I can work. Sometimes paying rent has been difficult; I have asked my parents for help, which I hate doing. I also miss things I enjoy—like the gym—because I am trying to study and work” (A7). |
| 29. Cannot work | “I cannot work, cannot maintain a relationship, and do not have much of a social life” (A2). |
| 30. Multiple job stress | “This time last year, after moving to Townsville, I missed my family and felt alone. The shift to trimesters increased workload. I also work in two pharmacies, so juggling everything was really difficult” (A7). |
| 31. Study and work anxiety | “Last year I felt alone and overwhelmed—anxious about work, study load, and assignments” (A7). |
| 32. Overwhelming transition | “Going straight from Grade 12 into anything medical was very overwhelming at first………” (A4). |
| 33. Content gap | “…………There is a lot you need to know that you do not get taught in school. Everything we have learned was only briefly touched on before, and now, in first year, it is “you need to know all this content before placement.” There is a strict cutoff on whether you are ready—yes or no—and there is not really an extension” (A4). |
| 34. Placement expectations | “…….Everything we have learned was only briefly touched on before, and now, in first year, it is “you need to know all this content before placement” (A4). |
| 35. Difficulty in adapting to university assessment style | “Academic challenges, I suppose, are just adapting to how the assessments come around. In school, it was always a very direct build up to exams, but here, already in week two” (A3). |
| 36. Difficulty in adapting to university culture | “In first year I did not really take uni seriously and failed a subject. It was my fault, but repeating meant I fell behind and would not be with my original friends. As the degree progressed it got harder—remembering everything we had already learned, learning new content, and keeping up with new regulations” (A7). |
| 37. Adjustment issues | “As the degree progressed it got harder—remembering everything we had already learned, learning new content, and keeping up with new regulations. I also started uni in a different city and moved to make it easier. Moving away from home was hard to adjust to, but I am doing better now” (A7). |
| 38. Lack of engagement | “In first year I did not really take uni seriously and failed a subject. It was my fault, but repeating meant I fell behind and would not be with my original friends. As the degree progressed it got harder remembering everything we had already learned, learning new content, and keeping up with new regulations” (A7) |
| 39. Falling behind peers | “In first year, I did not really take uni seriously and failed a subject. It was my fault, but repeating meant I fell behind and would not be with my original friends. As the degree progressed it got harder remembering everything we had already learned, learning new content, and keeping up with new regulations” (A7). |
| 40. First year stress | “I cannot say much as it is my first year, but it has definitely caused stress—especially with the early introduction of assignments. It just piled up quickly right from the start” (A3). |
| 41. Reduced social life | “Yes—during assessment blocks I am studying eight to ten hours a day. Social things become “eat with housemates, then back to study” (A1).  “I cannot work, cannot maintain a relationship, and do not have much of a social life” (A2).  “Socially, I have had to turn down opportunities with friends because of assignments or group meetings. I am on campus about four days a week” (A4).  “It means you cannot do everything. When that kind of week hits, I basically sit in my room studying almost 24/7. It makes you sacrifice opportunities and your social life—but for me it is mainly that week; you still have time outside it” (A5). |
| 42. Missing family events | “Yes—during assessment blocks I am studying eight to ten hours a day. Social things become “eat with housemates, then back to study.” I have missed family events, and sleep tends to slip in OSCE week if I am not careful” (A1) |
| 43. Reduced family and relationship time | “Definitely. I am a full-time student, so I allocate time to it every day—about six to eight hours between classes, tutorial preparation, and mandatory learning/certifications. It is time-consuming and takes away from relationships, family, and quality time” (A8). |
| 44. Relationship difficulty | “I cannot work, cannot maintain a relationship, and do not have much of a social life” (A2). |
| 45. Relationship strain | “In exam periods everything lands at once. I leave messages unread for days or weeks. People will say, “You have not responded in two weeks,” and I am like, “Yeah, I have been busy.” That can impact relationships, though most people understand” (A5). |
| 46. Sacrificing sleep | “By sacrificing. Mostly sleep and social time” (A2). |
| 47. Sacrificing social life | “By sacrificing. Mostly sleep and social time” (A2). |
| 48. Sacrificing personal life | “By sacrificing personal life—this semester I have been so stressed I have done nothing but uni. That means sacrificing self-care, exercise, and sleep to keep up” (A6).  “Mid-trimester, when everything is due, I sacrifice gym and personal time. I get up earlier to study and then go to work” (A7). |
| 49. Loss of self-care | “By sacrificing personal life—this semester I have been so stressed I have done nothing but uni. That means sacrificing self-care, exercise, and sleep to keep up” (A6). |
| 50. Social life integrated with university life | “I feel fortunate that a lot of my social life goes hand-in-hand with uni. I have made pharmacy friends and we share classes, so we socialise at uni or during long breaks—going into the city or the botanic gardens. That reduces how much “balancing” I need to do” (A4). |
| 51. Socialising with classmates | “I feel fortunate that a lot of my social life goes hand-in-hand with uni. I have made pharmacy friends and we share classes, so we socialise at uni or during long breaks—going into the city or the botanic gardens. That reduces how much “balancing” I need to do” (A4). |
| 52. Academics over social life | “I put academics first. Friends can postpone; you cannot postpone an exam—so I sacrifice social life for academics” (A5). |
| 53. Reduced social life impacting mental health | “Pharmacy content is challenging and mentally demanding. With friends, I rarely see them—maybe one friend every one to three months—because I barely have time. They are understanding, but it is consuming. Yes, it impacts mental health. I have a history of depression and generalised anxiety disorder, and the pressure can trigger that” (A8). |
| 54. Switching off difficulty | “Switching off after late shifts. It gets difficult for me to sleep and focus the next day” (A1). |
| 55. Financial stress | “The main challenge is financial. I come from a low- to medium-income household, and my parents cannot help with study fees, including mandatory placements” (A8).  “I do not get Centrelink or scholarships, so I have to support myself. Workload affects the hours I can work” (A7). |
| 56. Family unable to fund | “The main challenge is financial. I come from a low- to medium-income household, and my parents cannot help with study fees, including mandatory placements” (A8). |
| 57. Reduce income | “Socially, I have had to turn down opportunities with friends because of assignments or group meetings. I am on campus about four days a week. My workplace is closed Sunday and Monday—and those happen to be my two days off—so I have not been able to make as much income” (A4). |
| 58. Financial stress from failing | “I feel pressure to pass because my family helps where they can. I have been told I cannot fail a subject; that would mean an extra year of fees and an extra year of mandatory placement with no income” (A8). |
| 59. Paying own expenses | “I pay for my own expenses but not rent, since I live at home” (A2). |
| 60. Long commute | “Honestly, I am pretty privileged—nothing major comes to mind. The biggest thing is commuting…….. For a non-8 a.m. class I am leaving at 7 a.m.—about two hours each way, so roughly four hours a day” (A5). |
| 61. University’s location adds to travel burden | “Honestly, I am pretty privileged—nothing major comes to mind. The biggest thing is commuting………. For a non-8 a.m. class I am leaving at 7 a.m.—about two hours each way, so roughly four hours a day” (A5). |
| 62. Unrealistic program structure | “The program structure feels unrealistic and hard to balance; many in my cohort have struggled with the transition—though it is getting better” (A7). |
| 63. Increased stress and anxiety | “A significant increase in stress and anxiety. I had a history of anxiety that had settled, but it has flared up and now affects both my studies and personal life” (A6). |
| 64. Increase in stress | “A significant increase in stress and anxiety. I had a history of anxiety that had settled, but it has flared up and now affects both my studies and personal life” (A6). |
| 65. Anxiety impacting life and study | “A significant increase in stress and anxiety. I had a history of anxiety that had settled, but it has flared up and now affects both my studies and personal life” (A6). |
| 66. Mental health history triggered | “Pharmacy content is challenging and mentally demanding. With friends, I rarely see them—maybe one friend every one to three months—because I barely have time. They are understanding, but it is consuming. Yes, it impacts mental health. I have a history of depression and generalised anxiety disorder, and the pressure can trigger that” (A8). |
| 67. Felt lonely | “Last year I felt alone and overwhelmed—anxious about work, study load, and assignments” (A7). |
| 68. Emotional conflict | “There was a time in my studies when I felt conflicted with myself, also had prolonged sadness, confusion, loss of direction. I struggled to get up or do what I used to enjoy” (A8). |
| 69. Anxiety about multiple stressors | “………If anxiety becomes pervasive—worrying excessively about family health, finances, living situation, food at home—beyond my reasonable control, I seek help” (A8). |
| 70. Stress in busy times | “My family is proud and mainly wants me to finish on time and get a stable role. That is reasonable, but it is still pressure. Friends are in similar cycles; we catch up maybe monthly. Everyone gets it. I had sleeping difficulty in second year—due to overthinking. I am better now, but busy periods can still trigger it if I do not keep routines” (A1). |
| 71. Stress affecting functioning | “Severity and impact. I recently decided to seek help because stress was causing exhaustion, overwhelm, breakdowns, and affecting work and relationships. I contacted the university support team” (A6). |
| 72. Academics as priority | “It has always taken the forefront. I have always been focused on my school and academics. I prioritise them over family or work commitments—or at least try to” (A3). |
| 73. Study over family | “It has always taken the forefront. I have always been focused on my school and academics. I prioritise them over family or work commitments—or at least try to” (A3). |
| 74. Family pressure to become stable | “My family is proud and mainly wants me to finish on time and get a stable role. That is reasonable, but it is still pressure. Friends are in similar cycles; we catch up maybe monthly. Everyone gets it. I had sleeping difficulty in second year—due to overthinking. I am better now, but busy periods can still trigger it if I do not keep routines” (A1). |
| 75. Family expectation to succeed | “My family expects me to succeed and get a job. Friends from other degrees are shocked at how intense pharmacy is” (A2). |
| 76. Family has reasonably high expectations | “My family has a big impact, though it is not harsh. It is more a “do your best” approach, but still a reasonably high expectation. It pushes me to keep going because that is what is expected” (A3). |
| 77. High family expectations | “My family always expected uni—I was seen as a smart kid—so uni was the default. Friends’ kind of see me as “the smart one,” which adds peer pressure about how I am perceived if I fail” (A5).  “My mum is not strict, but she pushes for passing everything and good grades. I want to make my family proud……..” (A6). |
| 78. Family concerned about overstudying | “They are quite understanding and supportive. They do worry when I study constantly—home from uni, quick dinner, back to study—and that they do not see enough of me. If I am struggling, I tend to retreat. But overall, they understand some things are just difficult” (A4). |
| 79. Supportive family | “They are quite understanding and supportive. They do worry when I study constantly—home from uni, quick dinner, back to study—and that they do not see enough of me. If I am struggling, I tend to retreat. But overall, they understand some things are just difficult” (A4).  “I am lucky—no pressure for top grades. They just want me to try my best and pass. I call them every day, so I have that support. Pharmacy friends are very supportive; we study together and help each other” (A7). |
| 80. Family support | “I am lucky to have strong family support. I know not everyone has that. I have not had major problems, but I know many others do” (A3).  “I am fortunate to have an understanding family and partner. They know I cannot be there for everything” (A8).  “There is definitely a cultural idea that you should deal with it yourself—do not talk about it. That would be a barrier. For me, my family would be supportive, but I would not feel comfortable telling friends if I sought professional help” (A3). |
| 81. Family becoming more accepting | “Not very well. Getting help is not common “back home.” My immediate family (migrated from New Zealand) has come around after seeing me go through it” (A8).  “I think my family is open to the idea, but societal stigma is still there which makes it awkward” (A1). |
| 82. Parents and family as first support | “If I noticed I was not performing well at university or being absent-minded, that would push me to seek help. Also, if my sleep or eating was affected, I would take that as a sign. Definitely my parents or close family would be my first point of contact. I trust them most. I would talk it through with them, and then either we would figure it out together or they would help me find professional support” (A3). |
| 83. Positive expectations from family | “My mum is not strict, but she pushes for passing everything and good grades. I want to make my family proud……………………” (A6). |
| 84. House routine work | “We run a house roster and batch-cook on Sundays. My calendar has non-negotiable study blocks, rostered shifts, and actual rest. I also set tiny connection goals—like sending a voice note to Mum (A1). |
| 85. General household work | “I do not have many set responsibilities, just helping around the house when I can. My family has always been very supportive of my schooling” (A3).  “Recently caring for a dog who was dying; we put him down last Friday. It was shared across the family” (A5).  “I keep my spaces clean, but I also manage family finances (budgets), help with resumes and applications, and handle a lot of paperwork (super, mortgages, citizenship). I am the youngest and currently the only Australian citizen in my immediate family, so these tasks often fall to me and take time from study…..” (A8). |
| 86. Partner sharing household tasks | “I am fortunate to have an understanding family and partner. They know I cannot be there for everything. My partner takes on a lot—he does the grocery shopping; I rarely cook. I make about $500 a week and have bills, so I do not usually pay for groceries either” (A8). |
| 87. Sharing tasks with partner | “With my partner, we share daily household tasks—cleaning, laundry, cooking, paying bills—and caring for our pet” (A6). |
| 88. Tutoring family member | “I tutor a younger cousin each week” (A1). |
| 89. Daily family support | “I talk to my family every day and they help, but when I felt the way I did, professional help was what I needed (GP/counselling; the short medication course helped too)” (A7). |
| 90. Family support for familial issues | “Interpersonal, relationship, and study-related problems are usually addressed through professional support (including couples therapy). Familial issues are often handled within the family” (A8). |
| 91. Family support as coping | “Call my parents (and see our dogs on video), use a walking pad, exercise, talk to friends, and take complete breaks from uni when I am spiralling—distraction helps me reset” (A7). |
| 92. Managing household responsibilities | “I keep the house clean, take care of the dog, and make most dinners since I am usually home earlier than my parents. It influences timing…….” (A4). |
| 93. No family responsibilities | “It is just me—no specific responsibilities” (A7). |
| 94. Peer understanding | “My family is proud and mainly wants me to finish on time and get a stable role. That is reasonable, but it is still pressure. Friends are in similar cycles; we catch up maybe monthly. Everyone gets it. I had sleeping difficulty in second year—due to overthinking. I am better now, but busy periods can still trigger it if I do not keep routines” (A1). |
| 95. Academic competitiveness with friends | “My family has a big impact, though it is not harsh. It is more a “do your best” approach, but still a reasonably high expectation. It pushes me to keep going because that is what is expected. My friends are also quite academic, so there is competitiveness. I feel I have to maintain my character and keep achieving as I used to” (A3). |
| 96. Peer perception pressure | “Friends kind of see me as ‘the smart one’ which adds peer pressure about how I am perceived if I fail” (A5). |
| 97. Pressure not to fail | “I feel pressure to pass because my family helps where they can. I have been told I cannot fail a subject; that would mean an extra year of fees and an extra year of mandatory placement with no income” (A8). |
| 98. Comparisons with peers | “…….I also find myself comparing to friends in other degrees or the same degree” (A6). |
| 99. Friends surprised by pharmacy course intensity | “My family expects me to succeed and get a job. Friends from other degrees are shocked at how intense pharmacy is” (A2) |
| 100. Supportive friends | “Friends are a big support. I also think helplines like Lifeline are great—they are free, anonymous, and easy to access without having to commit to a formal appointment” (A3).  “I am lucky—no pressure for top grades. They just want me to try my best and pass. I call them every day, so I have that support. Pharmacy friends are very supportive; we study together and help each other. It is not competitive. Friends outside pharmacy are supportive too—I complain about uni and they encourage me” (A7). |
| 101. People having different coping strategies | “Everyone copes differently. I tell friends I am available to talk or grab lunch as a distraction. For me, distraction helps me come back with a fresher mind” (A7). |
| 102. Validation of others experiences | “I think your study is great. Mental health is central—people deal with things differently, and that is okay. No further comments” (A8). |
| 103. Pharmacy forced quitting sport | “I used to represent Australia in gymnastics. I managed it while studying dietetics, but pharmacy made me quit. That was upsetting” (A2). |
| 104. Pressure to maintain identity | “My family has a big impact, though it is not harsh. It is more a “do your best” approach, but still a reasonably high expectation. It pushes me to keep going because that is what is expected. My friends are also quite academic, so there is competitiveness. I feel I have to maintain my character and keep achieving as I used to” (A3). |
| 105. Get on with it | “My extended family is from New Zealand. Older relatives tend to “just get on with it.” They are not anti-help, but talking about feelings is not natural. My immediate family is more open” (A1). |
| 106. Just get on with it attitude | “My extended family is more “just get on with it.” My immediate family is more open to discussing feelings and help” (A1). |
| 107. Discomfort talking about feelings | “My extended family is from New Zealand. Older relatives tend to “just get on with it.” They are not anti-help, but talking about feelings is not natural. My immediate family is more open” (A1). |
| 108. Mental health not equal to physical health | “It is a bit strange. People say it is important, but it is not really treated the same as physical health. It is often more of a throwaway statement—“make sure you look after your mental health”—but there is not as much real focus on it. It feels a bit foreign, something different, not part of normal health conversations” (A3). |
| 109. Talking about mental health feels a bit foreign | “It is a bit strange. People say it is important, but it is not really treated the same as physical health. It is often more of a throwaway statement—“make sure you look after your mental health”—but there is not as much real focus on it. It feels a bit foreign, something different, not part of normal health conversations” (A3). |
| 110. Societal stigma | “I think my family is open to the idea, but societal stigma is still there which makes it awkward” (A1). |
| 111. Poor acceptance | “Not very well. Getting help is not common “back home.” My immediate family (migrated from New Zealand) has come around after seeing me go through it” (A8). |
| 112. Taboo | “Hesitant—it feels taboo and awkward. Some have not experienced similar things, and others feel their problems “are not big enough” to mention” (A8). |
| 113. Mental health not taken seriously | Often minimised—people say “it is not that bad,” “others have it worse,” or make light of it” (A6). |
| 114. Stigma still there | “Many are scared to reach out because they expect a negative reaction. It’s improving, but stigma is still there” (A6). |
| 115. Talking about mental health struggles is discouraged | “There is definitely a cultural idea that you should deal with it yourself—do not talk about it. That would be a barrier. For me, my family would be supportive, but I would not feel comfortable telling friends if I sought professional help” (A3). |
| 116. Expectation to handle issues alone | “There is definitely a cultural idea that you should deal with it yourself—do not talk about it. That would be a barrier. For me, my family would be supportive, but I would not feel comfortable telling friends if I sought professional help” (A3). |
| 117. Cultural non-recognition | “As a man, you are not expected to have mental health struggles. My South African heritage does not really acknowledge mental health” (A2).  “Culturally it’s still not the norm. It isn’t necessarily looked down on, but it’s hard for people to do” (A8). |
| 118. Generational difference | “In Australian culture there is definitely a generational difference. Tell an 80-year-old you are seeing a therapist, and they might see it as shameful. To people our age, if you say you are on antidepressants, they might often say, “Me too.” In older generation there is more stigma. In younger generation there is more acceptance” (A5). |
| 119. Stigma in older generation and more acceptance in younger | “In Australian culture there is definitely a generational difference. Tell an 80-year-old you are seeing a therapist, and they might see it as shameful. To people our age, if you say you are on antidepressants, they might often say, “Me too.” In older generation there is more stigma. In younger generation there is more acceptance” (A5). |
| 120. Generational openness | “My generation is more open and aware than older ones” (A2).  “Older guys (some pharmacists in their mid-30s) clearly struggle but will not talk. Younger students in their 20s are much more open. Some older folks say “we should be open” but do not apply it to themselves” (A5). |
| 121. Younger students more open | “Older guys (some pharmacists in their mid-30s) clearly struggle but will not talk. Younger students in their 20s are much more open. Some older folks say “we should be open” but do not apply it to themselves” (A5). |
| 122. Male stigma | “As a man, you are not expected to have mental health struggles. My South African heritage does not really acknowledge mental health” (A2). |
| 123. Medical students more aware | “Medical students are more aware of the mental health issues, I guess” (A3). |
| 124. Comfortable discussing mental health | “Comfortable. I treat it like talking about sleep hygiene or vaccinations” (A1). |
| 125. Comfortable discussing with close friends | “With my close friends, mostly okay. I would still be hesitant, but if I thought it was important for them to know, I would tell them” (A3).  “Very comfortable” (A7).  **“**Very comfortable. I am open to help others—share experiences, coping mechanisms, and remind them it is not their fault” (A8). |
| 126. Comfortable discussing with inner circle | “Very comfortable with a select few (partner, brother, close friends) and we support each other. With others, I am hesitant” (A6). |
| 127. Hesitant generally talking about mental health | “With my close friends, mostly okay. I would still be hesitant, but if I thought it was important for them to know, I would tell them. That way they would understand what I am going through and could support me” (A3). |
| 128. Hesitant with others | “Very comfortable with a select few (partner, brother, close friends) and we support each other. With others, I am hesitant” (A6). |
| 129. Not comfortable telling friends | “With my uni friends, they are still quite new, so I would not go very in-depth yet. They are aware of some challenges I have had. It is not necessarily fair on them—they are not trained—so I would prefer a professional support team” (A4).  “There is definitely a cultural idea that you should deal with it yourself—do not talk about it. That would be a barrier. For me, my family would be supportive, but I would not feel comfortable telling friends if I sought professional help” (A3). |
| 130. Limited openness | “Very comfortable with a select few (partner, brother, close friends) and we support each other. With others, I am hesitant” (A6). |
| 131. Mixed openness | “Mixed. Many are open; some feel their concerns “are not bad enough” (A1). |
| 132. General openness | “Yes, definitely” (A8). |
| 133. Personal limited sharing | “It should be open. The more it is talked about, the better—especially for people who need it. I personally do not share much about myself” (A6). |
| 134. Generally limited self-disclosure | “Depends how close I am to them. Some I fully trust and have told a lot; others I have not told anything. Generally, I will reveal a little, but I do not talk much about myself” (A5). |
| 135. Open communication valued | “It is a good thing that mental health is talked about in pharmacy. It is better to be open so everyone knows what to do if they are not okay, rather than keeping silent” (A3). |
| 136. Openness should be encouraged | “It should be open. The more it is talked about, the better—especially for people who need it. I personally do not share much about myself” (A5). |
| 137. Some friends value openness | “Some of my friends see it as important to talk about, while others are very guarded. For them, it is more of a personal issue, something to be kept within close family” (A3). |
| 138. Similar attitudes among peers | “We have not gone deep yet, but I believe we are fairly similar—open and safe” (A4). |
| 139. Similar attitude among close friends | “Not in my close circle—we are aligned” (A8). |
| 140. Close friends open | “Uni friends have not really discussed mental health with me; close friends outside uni have been more open about their struggles. So openness differs” (A6). |
| 141. University friends do not open to discuss | “Uni friends have not really discussed mental health with me; close friends outside uni have been more open about their struggles. So openness differs” (A6). |
| 142. Lack of shared experience | “Hesitant—it feels taboo and awkward. Some have not experienced similar things, and others feel their problems “are not big enough” to mention” (A8). |
| 143. Proactive support before exams | “To an extent. I am proactive about reaching out before exams” (A1). |
| 144. High awareness of peers struggling | “Very aware. I can identify when classmates are struggling—for example, one isolates herself completely, studies excessively, and admitted she feels lonely and stressed” (A2).  “Very aware. I am observant and approachable; people tend to open up. I am non-judgmental” (A4). |
| 145. Aware that peers struggle | “I do not know many people in my course yet, but I am aware that lots of students struggle. I never assume everyone is fine” (A3). |
| 146. Limited discussion on mental health | “It does not come up much. You notice it during high-stress moments—people crying in OSCEs or before presentations. I know what close friends go through; most people do not discuss it generally” (A5). |
| 147. Aware in high stress moments | “It does not come up much. You notice it during high-stress moments—people crying in OSCEs or before presentations. I know what close friends go through; most people do not discuss it generally” (A5). |
| 148. Limited awareness | “I do not know everyone’s situation, but given my own experience, I assume many are struggling—some getting help, some unable to” (A6). |
| 149. Low early awareness | “Early on I was not very aware of the toll. As I progressed—and from my own experience—my awareness grew” (A7). |
| 150. Leadership role increases awareness | “I try to be more aware. I am on the NAPSA committee, organise events, and make sure students know how to reach me (phone/social). I tell them early it is a tough course and to reach out. I may not know what they are facing at a given moment—that is their choice—but I work to build relationships so they can come to me if needed” (A8). |
| 151. Respecting privacy | “I tell them early it is a tough course and to reach out. I may not know what they are facing at a given moment—that is their choice—but I work to build relationships so they can come to me if needed” (A8). |
| 152. Relationship building for support | “I tell them early it is a tough course and to reach out. I may not know what they are facing at a given moment—that is their choice—but I work to build relationships so they can come to me if needed” (A8). |
| 153. Completed MHFA | “Mental Health First Aid” (A1).  “Mental Health First Aid” (A8). |
| 154. No training attended | “No, but I would be interested” (A2).  **“**Not in the past. But I have recently enrolled in a Mental Health First Aid course for pharmacy students. I have only just been accepted, so I have not started yet. I also engage with public talks on mental health” (A3).  **“**No. But I plan to in the future” (A4).  **“**No” (A6). |
| 155. Interested in training | “No, but I would be interested” (A2).  “No. But I plan to in the future” (A4). |
| 156. Recently enrolled in MHFA | “Not in the past. But I have recently enrolled in a Mental Health First Aid course for pharmacy students. I have only just been accepted, so I have not started yet. I also engage with public talks on mental health” (A3). |
| 157. Engaging in MH talks | “Not in the past. But I have recently enrolled in a Mental Health First Aid course for pharmacy students. I have only just been accepted, so I have not started yet. I also engage with public talks on mental health” (A3). |
| 158. Partial MHFA training done | “I did the online Mental Health First Aid component. The in-person session got cancelled for low numbers, rescheduled, then cancelled again—now tentatively week 8, but unclear if it will run” (A5). |
| 159. High school MH training | “In high school we did Teen Mental Health First Aid—recognising signs/symptoms and what to do. I do not recall anything similar at uni” (A7). |
| 160. Mental health literacy as a skill | “I think mental-health literacy is a clinical skill. Your study will help a lot” (A1). |
| 161. Support acknowledged | “As a first-year, it has not been touched much yet—probably because things are still simple and placements have not started. We have been told support is there and reminded periodically. One idea: posters on bathroom doors—people often use bathrooms to regroup; discrete information there could help” (A4). |
| 162. Professional support | “Professional support for sleep and anxiety; informal housemate debriefs for day-to-day stress; targeted feedback from preceptors for confidence” (A1).  “Psychological help—seeing a counsellor and psychologist” (A2).  “Therapist, hobbies, and talking to people” (A4).  **“**Professional help—counsellors/psychologists—because they have tools and training. Family/friends can only go so far” (A6).  **“**I talk to my family every day and they help, but when I felt the way I did, professional help was what I needed (GP/counselling; the short medication course helped too)” (A7).  **“**Both professional and informal, depending on the issue. Interpersonal, relationship, and study-related problems are usually addressed through professional support (including couples therapy). Familial issues are often handled within the family” (A8). |
| 163. Professional help preferred | “With my uni friends, they are still quite new, so I would not go very in-depth yet. They are aware of some challenges I have had. It is not necessarily fair on them—they are not trained—so I would prefer a professional support team” (A4). |
| 164. Professional support for relationship and academic issues | “Both professional and informal, depending on the issue. Interpersonal, relationship, and study-related problems are usually addressed through professional support (including couples therapy). Familial issues are often handled within the family” (A8). |
| 165. Professional help as coping | “Seeing a psychologist, taking medication, and accepting sacrifices like sleep and social life. Mostly, I just push through” (A2). |
| 166. Short-term medication | " Last year I felt alone and overwhelmed—anxious about work, study load, and assignments. Recognising I was not feeling like my usual self pushed me to seek help. I went to a GP, did online counselling, and took an antidepressant for about a month” (A7). |
| 167. Medication for coping | “Prescription medication (Ativan) helps most. Otherwise: video games, shower, sleep. I am still working on healthier coping skills” (A6). |
| 168. Counselling if available | “Professional help—counsellors/psychologists—because they have tools and training” (A6).  “Psychological help—seeing a counsellor and psychologist” (A2). |
| 169. Informal support | “Professional support for sleep and anxiety; informal housemate debriefs for day-to-day stress; targeted feedback from preceptors for confidence” (A1).  “Both professional and informal, depending on the issue” (A8). |
| 170. Feedback for confidence | “Professional support for sleep and anxiety; informal housemate debriefs for day-to-day stress; targeted feedback from preceptors for confidence” (A1). |
| 171. Helpline support | “Friends are a big support. I also think helplines like Lifeline are great—they are free, anonymous, and easy to access without having to commit to a formal appointment” (A3). |
| 172. Hobbies as coping | “Therapist, hobbies, and talking to people” (A4).  “Hobbies—crocheting, colouring—hands-on activities that calm me; also music and talking to people” (A4). |
| 173. Talking as coping | “Therapist, hobbies, and talking to people” (A4).  “Hobbies—crocheting, colouring—hands-on activities that calm me; also music and talking to people” (A4). |
| 174. Social support | “Call my parents (and see our dogs on video), use a walking pad, exercise, talk to friends, and take complete breaks from uni when I am spiralling—distraction helps me reset” (A7). |
| 175. External support | “…..Hospital, where I work, mental health is well supported” (A2). |
| 176. Reliance on friends for support | “Friends are a big support. I also think helplines like Lifeline are great—they are free, anonymous, and easy to access without having to commit to a formal appointment” (A3). |
| 177. Box breathing as coping strategy | “Box breathing before OSCEs, 90-minute focus blocks with a 10-minute walking break, and a three-line morning plan (must/should/can). Priorities are protein breakfast, hydration, and lights-out by 11” (A1).  “Box breathing during acute anxiety; make a to-do list/plan; push through when needed; take breaks, eat well, hydrate, and sleep—if I cannot focus, I sleep and try again tomorrow” (A8). |
| 178. Morning planning routine | “Box breathing before OSCEs, 90-minute focus blocks with a 10-minute walking break, and a three-line morning plan (must/should/can). Priorities are protein breakfast, hydration, and lights-out by 11” (A1). |
| 179. Healthy habits | “Box breathing before OSCEs, 90-minute focus blocks with a 10-minute walking break, and a three-line morning plan (must/should/can). Priorities are protein breakfast, hydration, and lights-out by 11” (A1).  “Box breathing during acute anxiety; make a to-do list/plan; push through when needed; take breaks, eat well, hydrate, and sleep—if I cannot focus, I sleep and try again tomorrow” (A8). |
| 180. Taking break from studies as coping | “Taking a break—walking my dog, spending time with family or friends, doing anything unrelated to the problem. It stops the “snowball effect” and helps me come back calmer” (A3). |
| 181. Music as coping | “Hobbies—crocheting, colouring—hands-on activities that calm me; also music and talking to people” (A4). |
| 182. Task oriented as coping | “I am action-oriented. Getting the task done—exam, assignment, OSCE—removes the stress” (A5). |
| 183. Gaming, shower and sleep as coping | “Prescription medication (Ativan) helps most. Otherwise: video games, shower, sleep. I am still working on healthier coping skills” (A6). |
| 184. Distraction coping | “Everyone copes differently. I tell friends I am available to talk or grab lunch as a distraction. For me, distraction helps me come back with a fresher mind” (A7). |
| 185. People having different coping strategies | “Everyone copes differently. I tell friends I am available to talk or grab lunch as a distraction. For me, distraction helps me come back with a fresher mind” (A7). |
| 186. Self-intervention strategy | “Taking a break—walking my dog, spending time with family or friends, doing anything unrelated to the problem. It stops the “snowball effect” and helps me come back calmer” (A3). |
| 187. If performance impacted due to low mood, sleep disturbance then I go for help | “If I am flat for more than two weeks, cannot sleep, or anxiety starts affecting my performance I book my GP. If basic routines fix it, I hold off” (A1). |
| 188. Routine coping as first step | “If I am flat for more than two weeks, cannot sleep, or anxiety starts affecting my performance I book my GP. If basic routines fix it, I hold off” (A1). |
| 189. Decline in performance pushes towards seeking help | “My performance is the main factor. If I noticed I was not performing well at university or being absent-minded, that would push me to seek help. Also, if my sleep or eating was affected, I would take that as a sign” (A3). |
| 190. Sleep and eating changes as sign | “My performance is the main factor. If I noticed I was not performing well at university or being absent-minded, that would push me to seek help. Also, if my sleep or eating was affected, I would take that as a sign” (A3). |
| 191. Risk of harm pushes towards help seeking | “I assess risk—if there is any possibility of harm to myself or others, or that intrusive thoughts could push unhealthy behaviour, I would reach out immediately. My therapist would be my first point of contact—then I would let my parents know” (A4). |
| 192. Severity | “It depends on severity and whether it affects daily functioning. If I could not get out of bed or function, I would seek help. If I am functioning normally, probably not. I would probably Google it or use the university website to find support” (A5). |
| 193. Seeking help if it is severe | “It depends on severity and whether it affects daily functioning. If I could not get out of bed or function, I would seek help. If I am functioning normally, probably not. I would probably Google it or use the university website to find support” (A5). |
| 194. Seek help early | “It depends on severity and whether it affects daily functioning. If I could not get out of bed or function, I would seek help. If I am functioning normally, probably not. I would probably Google it or use the university website to find support” (A3). |
| 195. Seek professional help early | “Sort a GP mental-health care plan early. Think of wellbeing like an immunisation schedule—small, regular doses” (A1). |
| 196. Don’t avoid assessments | “Do not defer assessments to avoid stress. Sit down, study, do it. Once you are confident, the stress drops—the “what if I fail?” fades” (A5). |
| 197. Confidence reduces stress | “Do not defer assessments to avoid stress. Sit down, study, do it. Once you are confident, the stress drops—the “what if I fail?” fades” (A5). |
| 198. First step improves wellbeing | “Reach out—to a psychologist, the university support team, a trusted friend, or even online. Taking that first step made me feel better, even before anything else changed” (A6). |
| 199. Reminding yourself challenges are temporary | “Try a simple daily affirmation (say it in the mirror). Remind yourself it is temporary; your experience is real even if others do not understand. Do not compare yourself; everyone has different cards. Give yourself a break—none of this makes you a bad person” (A8). |
| 200. Parents and family as first support | “Definitely my parents or close family would be my first point of contact. I trust them most. I would talk it through with them, and then either we would figure it out together or they would help me find professional support” (A3). |
| 201. At last, pursue professional help | “My performance is the main factor. If I noticed I was not performing well at university or being absent-minded, that would push me to seek help. Also, if my sleep or eating was affected, I would take that as a sign. Definitely my parents or close family would be my first point of contact. I trust them most. I would talk it through with them, and then either we would figure it out together or they would help me find professional support” (A3). |
| 202. Seeking professional help first | “I assess risk—if there is any possibility of harm to myself or others, or that intrusive thoughts could push unhealthy behaviour, I would reach out immediately. My therapist would be my first point of contact—then I would let my parents know” (A4). |
| 203. Using online sources as help | “It depends on severity and whether it affects daily functioning. If I could not get out of bed or function, I would seek help. If I am functioning normally, probably not. I would probably Google it or use the university website to find support” (A5). |
| 204. University support sought | “Severity and impact. I recently decided to seek help because stress was causing exhaustion, overwhelm, breakdowns, and affecting work and relationships. I contacted the university support team. Last semester I downplayed it and did not reach out—I wish I had earlier” (A6). |
| 205. Availing professional help | “Last year I felt alone and overwhelmed—anxious about work, study load, and assignments. Recognising I was not feeling like my usual self pushed me to seek help. I went to a GP, did online counselling, and took an antidepressant for about a month” (A7). |
| 206. Seek help early | “Do not let problems get too bad. Small struggles are normal, but if it starts affecting you seriously, talk to someone—friends, family, or a professional. There is no harm in seeking help” (A3). |
| 207. Reach out for help | “Reach out—there are so many resources now. There is always another stepping stone. Once you start getting help, it is amazing how much better you feel” (A4).  “Reach out—to a psychologist, the university support team, a trusted friend, or even online. Taking that first step made me feel better, even before anything else changed” (A6). |
| 208. Prior negative help-seeking experience | “I did seek help but received none, so now I keep things to myself” (A2). |
| 209. Reluctant to seek help now | “I did seek help but received none, so now I keep things to myself” (A2). |
| 210. Difficulty in asking for help | “Asking for help—accepting and communicating the support I need—has been hard” (A8). |
| 211. Cost barrier | “Time and cost. As a university student I am pressed for time, and costs are not always covered—so those are the big ones” (A5). |
| 212. Fear of negative reaction | “Many are scared to reach out because they expect a negative reaction. It’s improving, but stigma is still there” (A6). |
| 213. No cultural barrier | “Yes. I would go to my therapist or psychiatrist, and I would inform my parents so they are up to date and not left wondering” (A4). |
| 214. Stigma still there | “Many are scared to reach out because they expect a negative reaction. It’s improving, but stigma is still there” (A6). |
| 215. Good university support available | “Good—short-wait counselling, a 24/7 line, and temporary adjustments through the mental health support group” (A1).  “I did not use it at the time—I went through my GP—but I know free services are available for students” (A7).  **“**Adequate—approximately ten free sessions per year, emergency hotline for acute episodes, and guidance to community services” (A8). |
| 216. Well established university support | “It seems thorough and well established. At orientation, there was clear information on where to go if you had mental health concerns. It feels like a normal part of university. The clear publication and visibility of services are very helpful” (A3).  **“**They talk about it in classes, but the main way is the website hub—resources and referrals. It is private and self-directed; staff in the student help centre also know the pathways. I have not used it myself, so no big critique” (A5). |
| 217. Accessible and confidential university support | “I have not used it much—just disability/extension forms. From the emails, posters, and social media, it seems accessible, confidential, and safe” (A4). |
| 218. Supportive program | “None to add. My program is supportive: required self-care training sessions each trimester (coping skills, who to contact), and understanding coordinators who make accommodations” (A8). |
| 219. Inadequate university support | “Very poor. The timetable is overwhelming—classes every day, hours of pre-work, huge workload, and little understanding from lecturers” (A2).  “Student Success has been good (first appointment is tomorrow). Counselling felt hard to access—limited appointments, delays, seemed on-campus only; waiting two to three weeks did not feel helpful. For pharmacy students specifically, beyond some sessions, there is basically nothing targeted; I would like a long-term mentoring option” (A6). |
| 220. Lack of pharmacy specific support | “Student Success has been good (first appointment is tomorrow). Counselling felt hard to access—limited appointments, delays, seemed on-campus only; waiting two to three weeks did not feel helpful. For pharmacy students specifically, beyond some sessions, there is basically nothing targeted; I would like a long-term mentoring option” (A6). |
| 221. Improving university support | “Improve school psychologists/counsellors. I have heard stories where it did not feel confidential, which puts students off. Clarifying and strengthening confidentiality would help” (A4). |
| 222. Fail option for non-core subject | “I think a pass/fail option for one non-core unit during heavy placement blocks could reduce GPA anxiety” (A1). |
| 223. Reduce workload | “Reduce workload, restructure timetables, and improve lecturer understanding” (A2). |
| 224. Redesign academic structure | “Reduce workload, restructure timetables, and improve lecturer understanding” (A2). |
| 225. In person reminders of help available | “More reminders from lecturers—like short messages at the end of classes that mental health support is available. Right now, most information is online, but it should be mentioned in person too” (A3). |
| 226. Posters to promote support available | “As a first-year, it has not been touched much yet—probably because things are still simple and placements have not started. We have been told support is there and reminded periodically. One idea: posters on bathroom doors—people often use bathrooms to regroup; discrete information there could help” (A4). |
| 227. Targeted reminders in exam period about support available | “Focus promotion around exam time—send reminders when crunch periods hit” (A5). |
| 228. Mentor program | “A sustained mentor program (pairing first/second years with senior students or pharmacists), and easier access to lecturers/course coordinators. More frequent, accessible touchpoints” (A6). |
| 229. Regular wellbeing check-ins | “With the shift to trimesters (four years to three), the program feels condensed and hard to balance with work and placements, especially without financial support. Regular wellbeing check-ins; brief in-lecture prompts (weekly “Are you okay?” and resource links); staff training/refresher on mental-health awareness and approaching students compassionately” (A7). |
| 230. In-lecture prompts | “With the shift to trimesters (four years to three), the program feels condensed and hard to balance with work and placements, especially without financial support. Regular wellbeing check-ins; brief in-lecture prompts (weekly “Are you okay?” and resource links); staff training/refresher on mental-health awareness and approaching students compassionately” (A7). |
| 231. Staff training | “With the shift to trimesters (four years to three), the program feels condensed and hard to balance with work and placements, especially without financial support. Regular wellbeing check-ins; brief in-lecture prompts (weekly “Are you okay?” and resource links); staff training/refresher on mental-health awareness and approaching students compassionately” (A7). |
| 232. Need degree specific support | “Pharmacy is very hard and the stress is not discussed enough. Many are overwhelmed and mental health is affected; I wish there were more ways to get help specific to our degree” (A6). |
| 233. Pharmacy is hard | “Honestly—do not do pharmacy. It is too hard, and I regret it” (A2).  “Pharmacy is very hard and the stress is not discussed enough. Many are overwhelmed and mental health is affected; I wish there were more ways to get help specific to our degree” (A6). |
| 234. Pressure to maintain identity | “My family has a big impact, though it is not harsh. It is more a “do your best” approach, but still a reasonably high expectation. It pushes me to keep going because that is what is expected. My friends are also quite academic, so there is competitiveness. I feel I have to maintain my character and keep achieving as I used to” (A3). |
| 235. Pharmacy forced quitting sport | “I used to represent Australia in gymnastics. I managed it while studying dietetics, but pharmacy made me quit. That was upsetting” (A2). |
| 236. People having different coping strategies | “Everyone copes differently. I tell friends I am available to talk or grab lunch as a distraction. For me, distraction helps me come back with a fresher mind” (A7). |
| 237. Validation of others experiences | “I think your study is great. Mental health is central—people deal with things differently, and that is okay. No further comments” (A8). |
