# Supplementary material for: Contextual influences on mental health in pharmacy education: a comparative study across higher- and lower-middle-income settings
Source: Med Educ Online. 2026 Nov 5;31(1):2756743. doi: 10.1080/10872981.2026.2756743 (PMC13757898; doi:10.1080/10872981.2026.2756743)
Supplement: Supplementary file 4 [file ZMEO_A_2756743_SM0018.docx]

**Interview Topic Guide FOR RESEARCH PROJECT**

**Supplementary File S1:**

1. **Introduction:**
   - Briefly introduce myself and explain the purpose of the interview.
   - Remind participant of the Participant Information Sheet sent to them which has all the confidentiality information, digitally recorded information, data storage etc. Confirm eligibility criteria. Once participants have read it and are happy to proceed, obtain verbal consent.
2. **Warm-Up Questions:**
   - Demographics
   - Age range
   - Gender
   - Year of course
   - Years of experience (if working)
   - If yes to above, Role (manager, employee)
   - Pharmacy professional background (If any)
   - Why did you decide to pursue a degree in pharmacy?
   - Are you in a relationship?
   - What are your living arrangements?
   - Do you live alone or have family and friends here?
   - Are you self-funded or on a scholarship or receive study assistance?

**Section 1: Academic and Personal Challenges**

a) **Academic Pressure:**

**Main question**

- Can you share some experiences related to the academic challenges you've faced as a pharmacy student and how they have influenced your well-being?

**Follow-up/prompt questions**

- In what ways has your academic workload impacted your life, and have there been times when specific aspects of the program affected your mental health?

b) **Work-Life Balance/ Personal challenges:**

**Main question**

- How do you meet the demands of your academic responsibilities while managing your personal life? What have you found to be the most difficult aspects of maintaining a balance between your academic responsibilities and your personal life?

**Follow-up/prompt questions**

- Often students have personal challenges to deal with when they're trying to focus on their studies. Describe any personal challenges you have faced during your studies? How were these challenges dealt with?

c) **Social and Family Expectations and Responsibilities:**

**Main question**

- How do the expectations of family and friends influence your academic experiences and mental health?

**Follow-up/prompt questions**

- Can you describe any family responsibilities you have and how they interact with your academic life? How do they affect your mental wellbeing?

**Section 2: Cultural Influences**

a) **Cultural Perceptions and challenges:**

**Main question**

- What is your personal opinion of how mental health is perceived in your culture?

**Follow-up/prompt questions**

- What cultural challenges do you/would you face when seeking help for your mental health?

**Section 3: Mental Health Awareness and Attitudes**

a) **Awareness and attitude:**

**Main question**

- How comfortable do you feel discussing these mental health issues with your peers? What are your thoughts on the openness of students in your program to discuss mental health matters?

**Follow-up/prompt questions**

- How would you describe your awareness of mental health issues among your peers?
- What are the differences in attitudes toward mental health within your social circle?
- Have you attended any mental health awareness program/training?

**Section 4: Help-Seeking Behaviours**

a) **Help-Seeking:**

**Main question**

- What factors influence your decision to seek or not seek help for mental health challenges, and how do you approach finding the support you need?

**Follow-up/prompt questions**

- What kinds of help have you found to be most useful or accessible, whether it be professional counselling, support from friends and family, or other methods?

b) **Support Systems:**

**Main question**

- How do you view the support systems available at your university for mental health? What aspects of these systems have been helpful, or what improvements would you suggest?

**Section 5: Recommendations and Solutions**

a) **Suggestions for Improvement and Personal Strategies:**

**Main question**

- What changes do you think would improve mental health support for students in your program?

**Follow-up/prompt questions**

- What personal strategies have you found helpful in managing mental health?
- What advice would you give to other students facing similar challenges?

**Conclusion**

15. **Wrap-Up:**
    - Is there anything else you would like to share about your experiences with mental health and help-seeking behaviours?
    - Do you have any final thoughts or comments?

Stop the recording and ask for the participant’s email address for voucher and/or results summary if participant wishes.
